# Temporal BMP4 effects on mouse embryonic and extraembryonic development

**DOI:** 10.1038/s41586-024-07937-5

**Published:** 2024-09-18

**Authors:** Ron Hadas, Hernan Rubinstein, Markus Mittnenzweig, Yoav Mayshar, Raz Ben-Yair, Saifeng Cheng, Alejandro Aguilera-Castrejon, Netta Reines, Ayelet-Hashahar Orenbuch, Aviezer Lifshitz, Dong-Yuan Chen, Michael B. Elowitz, Magdalena Zernicka-Goetz, Jacob H. Hanna, Amos Tanay, Yonatan Stelzer

**Affiliations:** 1 Department of Molecular Cell Biology, Weizmann Institute of Science, Rehovot, Israel; 2 Department of Computer Science and Applied Mathematics and Department of Molecular Cell Biology, Weizmann Institute of Science, Rehovot, Israel; 3 Department of Molecular Genetics, Weizmann Institute of Science, Rehovot, Israel; 4 Division of Biology and Biological Engineering, California Institute of Technology, Pasadena, CA USA; 5 Mammalian Embryo and Stem Cell Group, Department of Physiology, Development and Neuroscience, University of Cambridge, Cambridge, UK

**Keywords:** Cell lineage, Differentiation, Stem cells

## Abstract

The developing placenta, which in mice originates through the extraembryonic ectoderm (ExE), is essential for mammalian embryonic development. Yet unbiased characterization of the differentiation dynamics of the ExE and its interactions with the embryo proper remains incomplete. Here we develop a temporal single-cell model of mouse gastrulation that maps continuous and parallel differentiation in embryonic and extraembryonic lineages. This is matched with a three-way perturbation approach to target signalling from the embryo proper, the ExE alone, or both. We show that ExE specification involves early spatial and transcriptional bifurcation of uncommitted ectoplacental cone cells and chorion progenitors. Early BMP4 signalling from chorion progenitors is required for proper differentiation of uncommitted ectoplacental cone cells and later for their specification towards trophoblast giant cells. We also find biphasic regulation by BMP4 in the embryo. The early ExE-originating BMP4 signal is necessary for proper mesoendoderm bifurcation and for allantois and primordial germ cell specification. However, commencing at embryonic day 7.5, embryo-derived BMP4 restricts the primordial germ cell pool size by favouring differentiation of their extraembryonic mesoderm precursors towards an allantois fate. ExE and embryonic tissues are therefore entangled in time, space and signalling axes, highlighting the importance of their integrated understanding and modelling in vivo and in vitro.

## Abstract (web summary)

Temporally dynamic extraembryonic and embryonic BMP4 signalling shapes mouse embryo lineage choices.

## Main

**Fig. 1:**
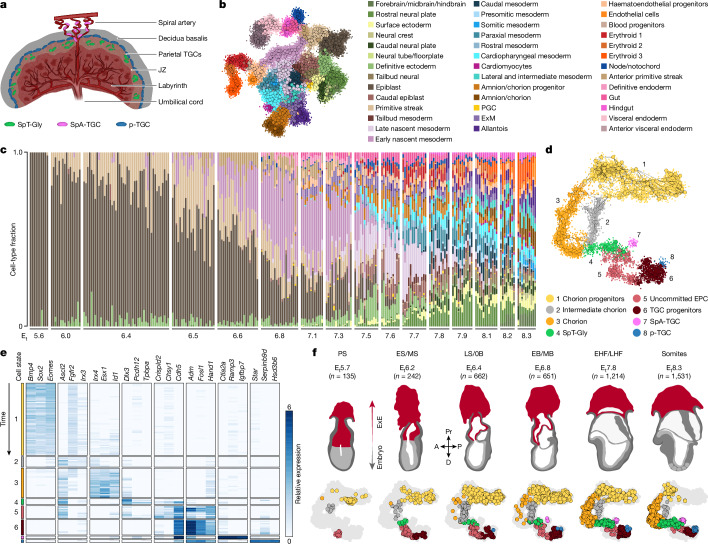
A unified extraembryonic–embryonic temporal model for gastrulation. **a**, Illustration of a mature mouse haemochorial placenta, showing the different subcompartments. SpA-TGC, spiral artery TGC; p-TGC, parietal TGC. **b**, UMAP (uniform manifold approximation and projection) of all embryonic and extraembryonic endoderm cells (*n* = 57,555 cells, excluding parietal endoderm). The small points represent single cells coloured by their cell states. The larger points represent metacells, connected through the edges to their most similar neighbours. Biological replicates were sampled over 43 experiments. **c**, The distribution of cell state composition per embryo (individual bar, *n* = 251 embryos), with embryos ordered by their transcriptional age and binned into 16 age groups annotated below by the mean estimated time (E_t_) of each age bin. **d**, UMAP of the ExE transcriptional manifold (*n* = 8,625 cells). **e**, The relative expression of cell-state-specific marker genes (log_2_[fold change] relative to the overall metacell average, negative values are not shown). Within each cell type, metacells are ordered by E_t_ (early to late; top to bottom). **f**, UMAPs of ExE cells (coloured points) over the entire ExE manifold (light grey points) corresponding to six age groups and their morphological illustration (top). A, anterior; D, distal; P, posterior; Pr, proximal. Source Data

The mammalian embryo develops alongside the placenta, a transient organ that constitutes the core infrastructure of the fetomaternal interface that nurtures the embryo until parturition through a constant supply of nutrients and gases, hormone production and immune modulation^1^. In mice, the embryo and its placenta develop in sync, emerging from the post-implantation blastocyst’s inner cell mass and their adjacent polar trophectoderm cells, respectively^2^. The latter gives rise to the ExE lineage, which differentiates to form the stereotypical structure of the mature placenta (Fig. 1a). The most proximal part of the ExE, termed the ectoplacental cone (EPC), gives rise to the outermost regions of the placenta, encompassing the parietal trophoblast giant cells (TGCs), spiral artery TGCs and the spongiotrophoblast and glycogen-accumulating (SpT-Gly) cells situated above and within the junctional zone (JZ)^1^. The innermost placental compartment, the labyrinth, constitutes the fetomaternal exchange apparatus. It comprises mostly syncytiotrophoblast cells descendant of the ExE chorion lineage, but also of dilated maternal sinuses, an exclusive TGC cell type^3^. The labyrinth also features fetal vessels lined by endothelial cells of mesodermal origin^4^, connected to the fetus through the umbilical cord. These structures trace their origins to the extraembryonic mesoderm (ExM) lineage.

In parallel to driving placenta development, the ExE compartment functions as a key early signalling centre for gastrulation. Indeed, proper patterning of the mouse embryo entails coordinated embryonic and EXE differentiation^5^. In one of the best-studied examples, cross-talk between BMP4 emanating from the ExE and NODAL from the epiblast orchestrates correct patterning of the primitive streak and promotes ExE development^6^. ExE-derived BMP4 signalling was further shown to be required for specification of posterior mesoderm, including primordial germ cells (PGCs)^6–8^. Additional cross-talks between ExE and embryonic lineages along the BMP axis or other pathways remain largely uncharacterized.

Thus far, challenges such as low cell frequency, intricate dissection procedures and potential biases related to non-canonical cell cycle and cell shape have hindered the construction of a precise temporal model during the early stages of placentation^6–9^, especially around the crucial gastrulation stage^3,10,11^. The complex temporal and spatial dynamics of the ExE-embryo interface also made it difficult to decouple their respective internal and intercompartment signalling function. Here we sought to map and time ExE cell states at the single-cell and single-embryo resolution, to model ExE lineage dynamics and to test the ExE-embryo signalling interface, particularly the time- and lineage-specific impact of BMP4 signalling.

## Temporal extraembryonic development

**Extended Data Fig. 1:**
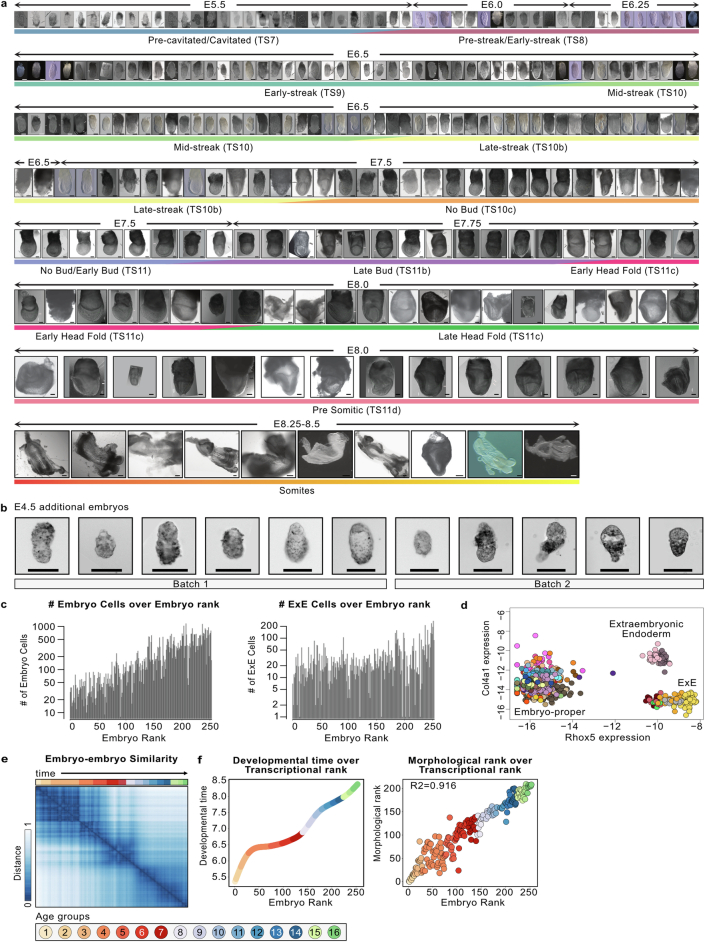
A single-embryo, single cell atlas of mouse gastrulation. **a**, Bright-field images of all embryos from E5.5-E8.5 used to construct the ExE-Embryo atlas (N_embryos_ = 251) ordered by their morphological rank, annotated by their dissected age (top, E#) and morphological stage (TS#; Theiler stages, bottom). Scale bar 100 µm. **b**, Bright-field images of two batches of flushed E4.5 blastocysts used for differential expression (Fig. 1g). Data represents 11 biologically independent samples over two experiments. Scale bar 100 µm. **c**, Embryonic (left) and ExE (right) cell count per embryo over each embryo’s transcriptional rank (p < 0.001, Wilcoxon rank test). **d**, Metacell gene-gene expression (log2 absolute expression) of Col4a1 over Rhox5 showing transcriptional separation of ExE from embryonic and extraembryonic endoderm cell states. **e**, Embryo-embryo transcriptional similarity matrix, embryos are annotated based on embryonic age groups (see legend below, same as in Fig. 1c). **f**, Developmental time (E_t_, left) and morphological rank (right) over embryo rank, annotated by age group. Source Data

**Extended Data Fig. 2:**
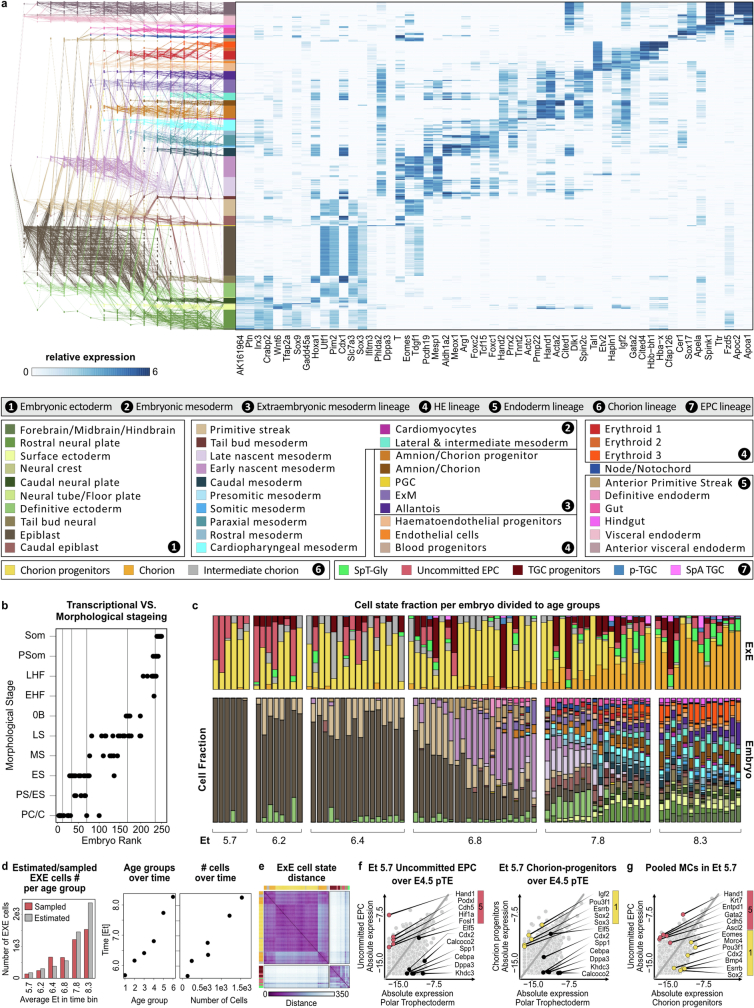
A unified extraembryonic/embryonic time-resolved model for gastrulation. **a**, Embryonic network flow model of differentiation (left). The model consists of metacells (nodes in rows, corresponding to the 16 embryonic age groups in Fig. 1c) distributed over E_t_ (x axis), and flows (edges) linking metacells between adjacent age groups. The first time point represents an artificial common source for all metacells. Right, Marker heatmap of relative expression (log2 scale, negative values are not shown) with representative feature genes for each cell state. See colour legend below. **b**, Morphological stage of individual embryos (black dots) over embryo rank, black lines represent ExE age group borders (n = 6). **c**, ExE (top) and embryonic (bottom) cell-state frequencies for individual embryos [N_embryos_ = 83] ordered by their transcriptional rank and binned into six ExE age groups. Each age group is annotated by its mean age (E_t_, bottom). **d**, Estimated (grey) and sampled (red) number of ExE cells per age group (left panel, see Methods), each age group is annotated by its mean age (E_t_, bottom). Estimated developmental time (E_t_) over ExE age groups (middle panel) and number of cells per ExE age group (right panel). **e** Metacell-metacell distance matrix across ExE metacells. Shown is logistic distance between absolute expression profiles of feature genes (558 genes). **f-g**, Differential gene expression between E4.5 polar trophectoderm (pTE) cells, isolated from a pool of 11 blastocysts (pTE enriched genes marked with black points) and the two earliest (mean E_t_ = 5.7) metacell populations of the ExE; Uncommitted EPC (left, EPC marker genes highlighted in light red) and chorion progenitors (right, chorion progenitor marker genes highlighted in yellow). Source Data

To map parallel transcriptional dynamics in embryonic and ExE lineages, we assembled data on around 68,000 single cells derived from 287 individually processed embryos alongside their extraembryonic tissues. Embryos were sampled from the egg cylinder stage to early somitogenesis, with additional two batches of flushed embryonic day 4.5 (E4.5) blastocysts (Extended Data Fig. 1a,b). We noted that ExE cells (overall, *n* = 8,652) present a fluctuating fraction of all sampled cells over time (Extended Data Fig. 1c), and concluded that timing individual ExE samples is unreliable. To circumvent this, we used the embryo proper as a scaffold, expanding our previous temporal model for embryonic gastrulation^12^ (Fig. 1b,c and Extended Data Figs. 1d–f and 2a) and assigning a time reference for ExE cells on the basis of the time label of their corresponding embryo (denoted as E_t_, indicating relative developmental timing; see Methods).

We annotated the ExE transcriptional manifold (Fig. 1d,e) and defined three progenitor-like cell states: chorion progenitor cells, uncommitted EPC cells and TGC progenitors. We also annotated states linked with chorion cells, prospective JZ cells (SpT-Gly cells), spiral-artery-associated TGCs and parietal TGCs. Notably, we also identified a cell population that largely repressed chorion progenitor markers but only partially activated the mature chorion program, which we termed intermediate chorion cells (Fig. 1d,e).

## Asynchronous differentiation of EPC cells

We traced ExE differentiation over six time bins that were aligned with the reference embryonic process (Fig. 1f and Extended Data Fig. 2b–d). We noted the distinct appearance of chorion progenitors and uncommitted EPC cells already at E_t_5.7 post-implantation embryos (Fig. 1f and Extended Data Fig. 2e), quantifying their transcriptional states using a metacell model^13,14^. Both programs repressed trophectoderm genes (*Dppa3*, *Calcoco2*, *Spp1* and *Cdx2*) compared with trophectoderm cells from E4.5 blastocysts (Extended Data Fig. 2f). Chorion progenitors upregulated several genes encoding transcription factors (TFs) and signalling effectors (*Eomes*, *Esrrb*, *Sox2*, *Pou3f1* and *Bmp4*), resembling previously described in vitro cultured trophoblast stem cells (TSCs)^15,16^. Uncommitted EPC cells robustly downregulate TSC-like signature genes and express different sets of genes encoding TFs and cell surface proteins (*Ascl2*, *Hand1*, *Cdh5* and *Entpd1*)^10,17,18^ (Fig. 1e and Extended Data Fig. 2g).

**Fig. 2:**
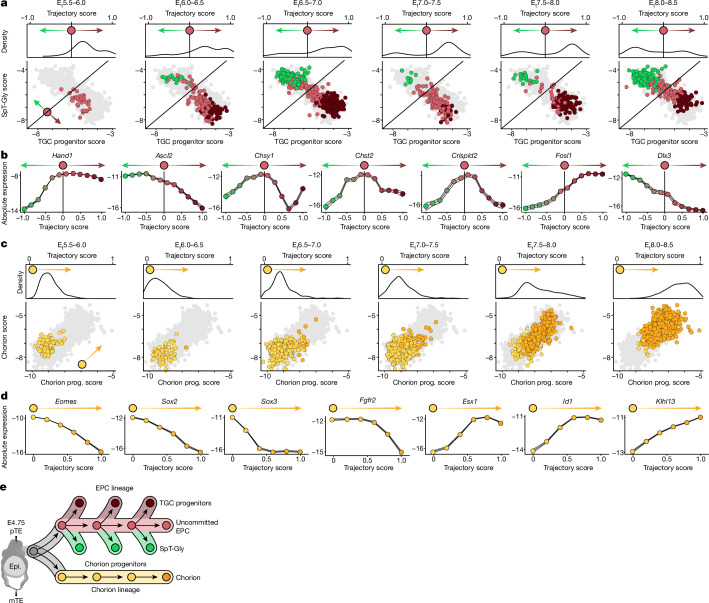
Differentiation dynamics in the ExE lineages. **a**, Aggregated single-cell expression of SpT-Gly cell-enriched genes (*n* = 98 genes; SpT-Gly score, *y* axis) over TGC-progenitor-enriched genes (*n* = 130 genes; TGC progenitor score, *x* axis) for all cells from the EPC lineage (light grey points), shown separately for a series of six time bins with 0.5 E_t_ intervals. Cells are coloured by their cell state. The top part of each panel shows the distribution of cells along the principal curve interpolating between TGC progenitors, uncommitted EPC and SpT-Gly cells (Methods and Extended Data Fig. 3b). The horizontal line (top) and diagonal line (bottom) intersects the principal curve at its midpoint; the slope of the diagonal was set at 1 for visualization purposes. **b**, Gene expression (log_2_[absolute expression]) of lineage-characteristic genes along the pseudotime trajectory interpolating TGC progenitors and SpT-Gly cells; the black line shows the middle reference point. **c**, Aggregated single-cell expression of chorion progenitor (prog.) enriched genes (*n* = 60 genes, chorion progenitor score, *y* axis) over chorion-enriched genes (*n* = 41 genes, chorion score, *x* axis) for all chorion progenitor and chorion cells (light grey points), as presented in **a** (the chorion lineage trajectory is shown in Extended Data Fig. 4b). **d**, Gene expression (log_2_[absolute expression]) of lineage-characteristic genes along the trajectory interpolating chorion progenitors and chorion. **e**, A suggested model of ExE differentiation dynamics. Epi., epiblast; mTE, mural trophectoderm; pTE, polar trophectoderm. Source Data

**Extended Data Fig. 3:**
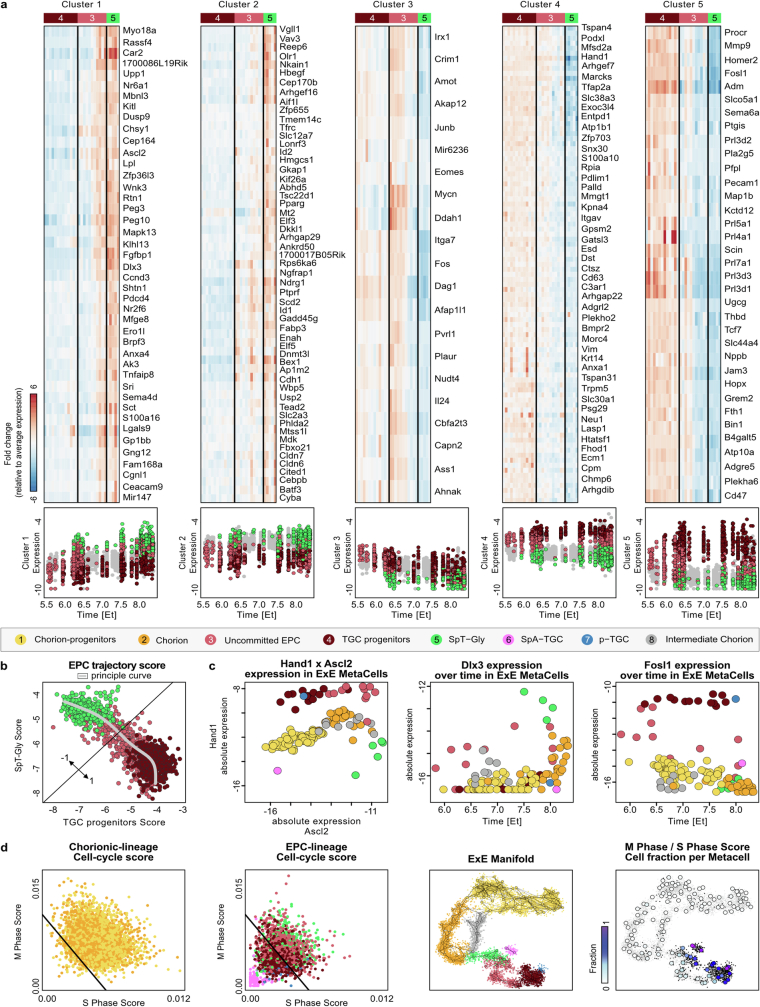
Differentiation dynamics of uncommitted EPC cells. **a**, Heatmaps of k-means clusters (k = 5) depicting relative expression (log2 fold change) of variable genes over EPC lineage metacells (top). Within each cell type, metacells are ordered by E_t_ (early-to-late; left-to-right). Aggregated single-cell expression from all genes per cluster (log2 absolute expression) over E_t_ (bottom). **b**, Principle curve (bold solid grey line) fitted to the joint distribution of single-cell SpT-Gly and TGC progenitor scores, and parameterized by values from −1 (high SpT-Gly score) to 1 (high TGC progenitor score). Intersection of the black line with the curve marks the midpoint on the curve. **c**, ExE metacell expression (log2 absolute expression) of Hand1 vs. Ascl2 (left), Dlx3 over transcriptional time (E_t_, middle) and Eomes over transcriptional time (E_t_, left). **d**, Cell-cycle analysis of ExE cellular states. Aggregated expression from mitosis-related genes (M-phase score, Methods) over DNA-synthesis-related genes (S-phase score) in the chorion lineage (far- Black rectangle; solid black line represents a threshold, with cells that fall below it classified as slowly cycling cells. For comparison, annotated ExE UMAP (right) and the Metacell fraction of slowly cycling cells (far-right), with single cells (small points) coloured by their cell cycle activity (black – slowly cycling cells, grey – actively cycling cells). Source Data

Within EPC states, the data indicated two main expression dynamics corresponding to SpT-Gly or TGC progenitor genes (clusters 1–2 versus clusters 4–5; Extended Data Fig. 3a). A smaller group involved EPC genes that decreased over time in all three cell types of the EPC lineage (cluster 3; Extended Data Fig. 3a). To characterize the dynamics of the two main programs, we computed a TGC progenitor and SpT-Gly score for each cell by summing the expression of genes in each program (Methods) and inferred their joint single-cell distribution over six embryonic time bins (Fig. 2a and Extended Data Fig. 3b). This showed that, at around E_t_5.5–6.0, only uncommitted EPC cells are observed, but starting at E_t_6.0, a continuum of TGC progenitors and SpT-Gly cell states arises. These results suggest that uncommitted EPC cells are maintained as a progenitor population over 3 days, with continuous bifurcation towards the differentiated progenies.

The kinetics of gene expression over the EPC differentiation trajectories suggested that the multipotency of the uncommitted EPC state is associated with co-expression of both *Hand1* and *Ascl2*, and that differentiation involves asymmetric repression of *Hand1* in SpT-Gly cells (approximately 12-fold) and of *Ascl2* in TGC progenitors (approximately 7-fold) (Fig. 2b and Extended Data Fig. 3c). Very few genes show a reduction in high expression levels in EPC cells towards the two fates (for example, *Chsy1*, *Chst2* and *Crispld2*). Medium-level expression of additional TF-encoding genes in uncommitted EPC cells, in particular *Dlx3* and *Fosl1*, is resolved asymmetrically through induction towards one fate and repression to the other (*Fosl1* is eventually active in TGC progenitors, and *Dlx3* is eventually active in SpT-Gly cells; Fig. 2b and Extended Data Fig. 3c). Commitment ultimately results in the induction of bona fide functional genes, such as those encoding prolactin units (such as *Prl3d1*)^19^, components of the fetomaternal interface (such as *Adm* and *Pecam1*) for TGC, or metabolite catalysis- and transport-related molecules (for example, *Car2* and *Sct*) for SpT-Gly cells (Extended Data Fig. 3a).

Two specialized TGC cell types, spiral-artery-associated TGCs and parietal TGCs, are shown to emerge as early as E_t_6.8 (Supplementary Table 2), representing the initiation of a process previously termed ‘vascular invasion’ and ‘endothelial mimicry’^1,19^. Profiling cell cycle gene expression for all ExE cells (Methods) showed that proliferative programs are observed pervasively throughout all states. The notable exception to this rule are TGCs and, more specifically, their progenies (Extended Data Fig. 3d). The resulting model points towards a cell cycle arrest process concomitantly with TGC maturation and later their physical invasion into the maternal tissue. At the same time, all of the other ExE cell states proliferate and expand rapidly. Together, these data show that uncommitted EPC cells are maintained as a progenitor population that continuously bifurcates through flexible combinatorial modulation of lineage-specific TFs.

## Differentiation of chorion progenitors

**Extended Data Fig. 4:**
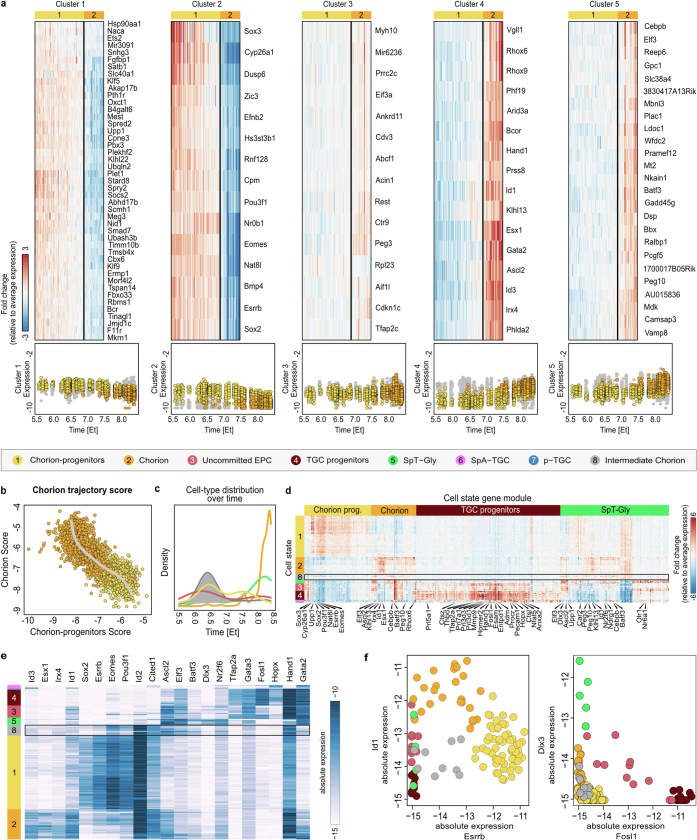
Differentiation dynamics of chorion progenitor cells. **a**, Heatmaps of k-means clusters (k = 5) of relative expression (log2 fold change) of variable genes over chorion lineage metacells (top). Within each cell type metacells are ordered by E_t_ (early-to-late; left-to-right). Aggregated single-cell expression from all genes per cluster (log2 absolute expression) over E_t_ (bottom). **b**, Principle curve (bold solid grey line) fitted to the joint distribution of single-cell chorion progenitor and chorion scores, parameterized by values from 0 (high chorion progenitors score) to 1 (high chorion score). **c**, Time distribution of ExE cell types. **d**, Heatmap showing ExE metacell expression (log2 fold change) over cell state markers associated with TGC progenitor, SpT-Gly, chorion progenitor, and chorion. Black rectangle highlighting intermediate chorion metacells. **e**, Heatmap of ExE metacell expression (log2 absolute expression) over a set of highly expressed transcription factors (TFs), with intermediate chorion metacells (black rectangle) displaying a unique combination of expressed TFs. **f**, ExE metacell gene-gene expression (log2 absolute expression) of Id1-Esrrb (left) and Dlx3-Fosl1 (right). Source Data

In contrast to the EPC cells, chorion progenitors show a simpler dynamic (Fig. 2c) involving a switch between a progenitor program defined by co-expression of genes encoding TFs including *Sox2*, *Pou3f1*, *Esrrb* and *Eomes* (clusters 1 and 2 in Extended Data Fig. 4a), and a differentiated chorionic program dominated by genes encoding TFs such as *Ascl2*, *Esx1*, *Gata2*, *Irx4*, *Id1* and *Id3* (clusters 4–5 in Extended Data Fig. 4a). This switch is gradual, with spurious initiation already observed at E_t_6.4, followed by the robust emergence of chorion cells at E_t_7.5, in sync with the rapid exhaustion of the progenitor population (Figs. 1f and 2c). Notably, ExE-derived chorion starts to appear at the same time as ExM-derived amnion/chorion, towards the end of E_t_6.8 (Extended Data Fig. 2c). Analysis of expression kinetics along the chorionic trajectory (Fig. 2d, Extended Data Fig. 4b and Supplementary Table 3) shows a gradual decline in genes encoding progenitor-associated TFs as cells intensify their chorionic signature (for example, *Sox3*). *Fgfr2* (previously linked with chorionic identities^20^) is maintained at high expression levels before rapidly declining with chorion differentiation at around E_t_7.5. Induction of chorion TF-encoding genes such as *Esx1* and *Id1* to maximal levels probably drives the more gradual increase of bona fide chorion markers such as *Rhox6*^21^. Notably, an early (approximately E_t_6.0; Extended Data Fig. 4c) population of cells that we annotated as intermediate chorion seems to largely repress progenitor TFs (*Eomes*, *Esrrb* and *Sox2*), and partially induce some chorion-associated genes (*Cited1*, *Ascl2* and *Id2*; Fig. 1e and Extended Data Fig. 4d,e). Intermediate chorion cells show clear separation from uncommitted EPC cells (low expression of *Dlx3* and *Fosl1*; Extended Data Fig. 4f), but their bipotency cannot be excluded at this point. In summary, chorion differentiation involves the coordinated repression of multiple progenitor TFs and induction of the functional chorionic program. This process occurs concomitantly with the continuous differentiation of uncommitted EPC cells (Fig. 2e).

## Spatial ExE differentiation

**Fig. 3:**
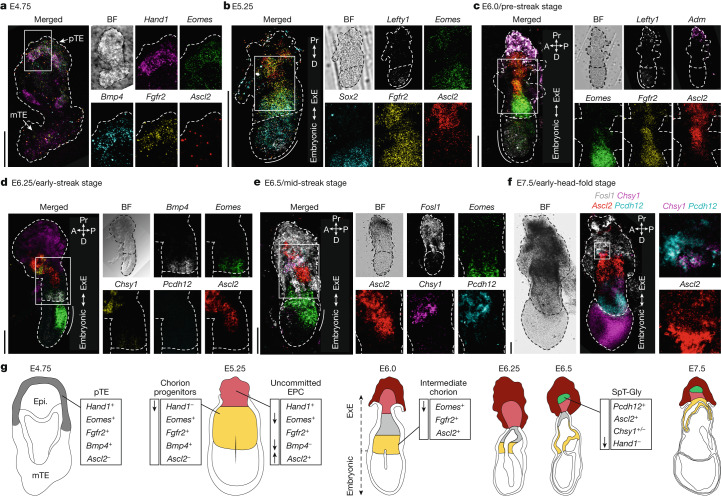
Spatiotemporal analysis of ExE cell states using combinatorial gene staining. **a**–**f**, Representative images of combinatorial mRNA molecule detection (hybridization chain reaction RNA fluorescence in situ hybridization (HCR–RNA-FISH; Methods; *n* = 3 (E4.75), *n* = 2 (E5.0), *n* = 3 (E5.25), *n* = 1 (E5.5), *n* = 1 (E5.75), *n* = 1 (E6.0), *n* = 4 (E6.25), *n* = 4 (E6.5), *n* = 1 (E7.5)) in a time series of embryos at E4.75 (**a**), E5.25 (**b**), E6.0 (**c**), E6.25 (**d**), E6.5 (**e**) and E7.5 (**f**). The areas indicated by white boxes are magnified on the right. For **a**–**f**, scale bars, 100 µm. The white dashed lines mark the embryo borders. The white solid lines mark the embryonic–ExE border (labelled and oriented with a white double head arrow legend to the right). Embryo axis: anterior (A), proximal (Pr), posterior (P) and distal (D). BF, bright field. In **c** and **d**, the distal/anterior visceral endoderm is marked by a solid white bold line in the merged image and *Lefty1* channel. In **e** and **f**, the primitive streak extension is marked by a solid white bold line in the merged image. **g**, An illustration summarizing the spatial location of cell state over developmental stages (chorion and chorion progenitors are coloured in yellow).

**Extended Data Fig. 5:**
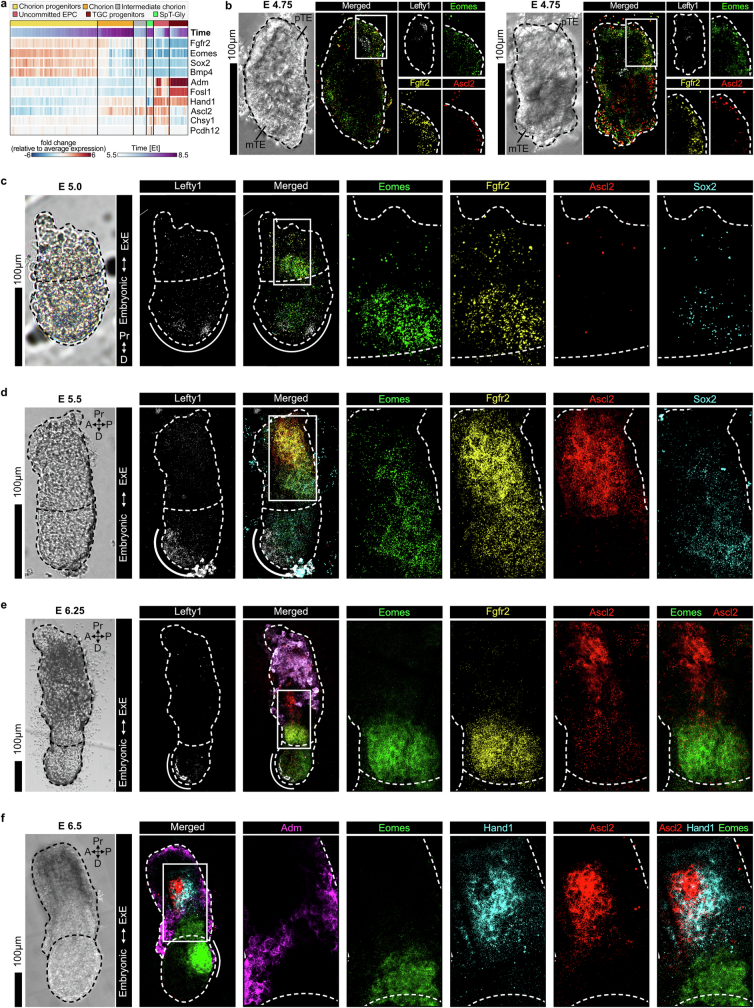
Spatiotemporal distribution of marker genes during ExE differentiation. **a**, Heatmap showing relative expression of cell state markers among ExE metacells (see legend at the top). Within each cell type metacells are ordered by E_t_ (early-to-late; left-to-right). **b-f**, Representative images of combinatorial HCR-RNA-FISH marker staining in embryos from E4.75 (a, N = 3), E5.0 (b, N = 2), E5.5 (c, N = 1), E6.25 (d, N = 4), and E6.5 (e, N = 4). All biologically independent samples over three experiments. Magnified regions are highlighted with a white solid line rectangle in the merged images. White dashed lines mark the embryo borders and the Embryonic-ExE border (oriented with a white double-head arrow). In panels **b-d**, the distal/anterior visceral endoderm is marked by a solid white bold line in the merged image and Lefty1 channel. Embryo axes; Anterior (A), Proximal (Pr), Posterior (P) and Distal (D). Bright field; BF. Source Data

To embed the new temporal ExE model within a spatial context, we used multiplexed in situ hybridization^22^ (Methods) with probes for genes with distinct lineage kinetics (Extended Data Fig. 5a). As the blastocyst matures to initiate the process of implantation, the differentiation of trophoblasts is influenced by their vicinity to the inner cell mass^15^. In this process, mural trophectoderm cells attach, implant and differentiate to primary TGCs within the decidualizing endometrium, while polar trophectoderm cells constitute the common progenitor for all ExE cells^16^. Spatial analysis of E4.75 embryos showed that polar trophectoderm cells concomitantly express both chorion progenitor (*Eomes, Fgfr2* and *Bmp4*) and uncommitted EPC (*Hand1*) markers (Fig. 3a and Extended Data Fig. 5b,c). We first observed clear spatial segregation between the two lineages along the proximal–distal axis at E5.25, when *Lefty1* marks the visceral endoderm at the most distal tip of the embryo (Fig. 3b). Chorion progenitor cells reside at the distal region of the ExE expressing *Eomes*, *Sox2* and *Fgfr2*, whereas an EPC-like cell population is located at the most proximal part, inducing *Ascl2* and reducing *Eomes* together with complete repression of *Sox2* (Fig. 3b and Extended Data Fig. 5d).

At E6.0–6.25 (Fig. 3c), we detected four populations with distinct ExE signatures distributed over the proximal–distal axis. From distal-to-proximal: chorion progenitors expressing *Fgfr2* and high levels of *Eomes*, followed by a population of cells expressing *Fgfr2*, *Ascl2* but with low levels of *Eomes*, corresponding to the intermediate chorion signature; more proximally, the uncommitted EPC population is marked by high *Ascl2*, reduced *Fgfr2* and a lack of *Eomes* expression, followed by the first bona fide *Adm*-expressing TGC progenitors. Taken together, these data confirm the model of early chorion and EPC separation and its specific spatial organization.

At the onset of gastrulation, coinciding with the induction of *Eomes* at the posterior side of the embryo (primitive streak), more distinct niches emerge within the ExE (Fig. 3d). The distal ExE niche contains chorion progenitor cells expressing *Bmp4* and *Eomes*. More proximally, we detect co-expression of *Ascl2* and *Chsy1* characteristic of uncommitted EPC cells. We could not detect *Pcdh12* transcripts in *Ascl2*-expressing cells, while marked expression of *Adm* is already detected in TGC progenitors at the most proximal tip of the ExE (Fig. 3d and Extended Data Fig. 5e). Together, this supports the notion that commitment towards TGC fates precedes SpT-Gly differentiation. At E6.5, we identify the first SpT-Gly cells expressing *Pcdh12*, an early marker for glycogen accumulating trophoblasts^23^ together with *Ascl2* and *Chsy1*. These cells seem to emerge from more-proximal *Ascl2*-expressing cells that do not express *Eomes*, and begin repressing *Chsy1* and *Hand1*, favouring the hypothesis that they are direct descendants of the EPC lineages and not intermediate chorion (Fig. 3e and Extended Data Fig. 5f). Finally, at E7.5, the chorion displays a characteristic folded structure marked by *Ascl2* while the EPC maintains the stratified architecture with a proximal-to-distal distribution of TGC progenitors and SpT-Gly cells, emerging from a population of EPC cells expressing *Chsy1* and *Ascl2* (Fig. 3f). Overall, these data outline a pattern of trophoblast differentiation along the proximal–distal axis. The continuous growth of the ExE reduces the ability of EPC cells to interact with the embryo proper, alongside increasing exposure to the decidual environment at the embryo-maternal interface (Fig. 3g).

## ExE–maternal and internal signalling cues

**Extended Data Fig. 6:**
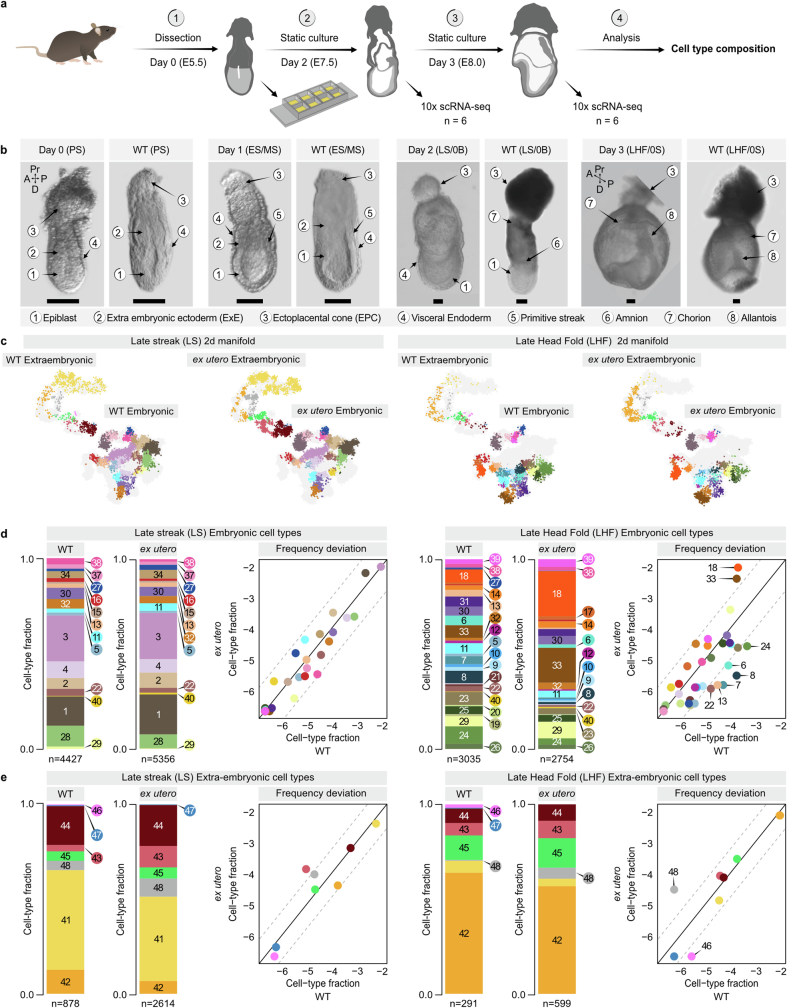
Single-cell analysis of ExE specification in ex utero cultured embryos. **a**, Scheme describing the experimental design for evaluating the transcriptome profile of ex utero cultured embryos. **b**, Representative bright-field images of embryos during ex utero culture (left, N = 12) and WT embryos (right, N = 12) at four different time points. All biologically independent samples over two experiments. PS – pre-streak; ES – Early streak; MS – Mid streak. Embryo axes; Anterior (A), Proximal (Pr), Posterior (P), and Distal (D). Scale bar 100 µm. **c**, Single-cell atlas projection of cells from ex utero embryos (right) and time-matched WT embryos (left). Shown are projections for late streak stage (left panel) and late head fold stage (right panel) for each group. Cells are coloured according to their projected cell state. See legend in Extended Data Fig. 2. **d-e**, pooled embryonic (d) and ExE (e) cell-state frequencies of time-matched WT embryos (left bars) and ex utero embryos (right bars) at the late streak (LS, left panel) and late head fold (LHF, right panel) stages. The left side of each panel displays the pooled frequency (in log2 scale) of cell types in ex utero embryos compared to time-matched WT embryos. The solid and dashed lines represent the x = y diagonal and a two-fold difference threshold, respectively. A numerical annotation indicates cell types that differ by more than two-fold. Source Data

To test the effect of the fetomaternal interface on ExE development, we cultured embryos dissected at E5.5 for 3 days using a recently described ex utero culture system^24^ (Extended Data Fig. 6a). The developmental progression of the embryonic compartment was highly similar between ex utero and in utero embryos. However, the size and morphology of the ExE compartment in most ex utero sampled embryos after 2 days of culture deviated from their wild-type (WT) counterparts after gastrulation (Extended Data Fig. 6b), possibly correlated with the relatively reduced (approximately 20%) efficiency of E5.5 embryos to develop normally until E11.0^24^. Using morphology selection, we assembled two cohorts of pooled embryos from day 2 (late streak stage) and day 3 (late head fold stage) ex utero cultures. Analysis using single-cell RNA-sequencing (scRNA-seq) showed that the embryonic compartment is highly conserved, with similar compositions for the ex utero and time-matched WT embryos (Extended Data Fig. 6c,d). Some possible increases in the amnion/chorion and erythrocyte frequencies in the later stages may be attributed to the differences in the dissection process in vivo and ex utero. Notably, ex utero cells were also robustly mapped to nearly all ExE cell states (Extended Data Fig. 6e), reflecting the autoregulatory capacity of the developing ExE even without a fetomaternal interface.

**Extended Data Fig. 7:**
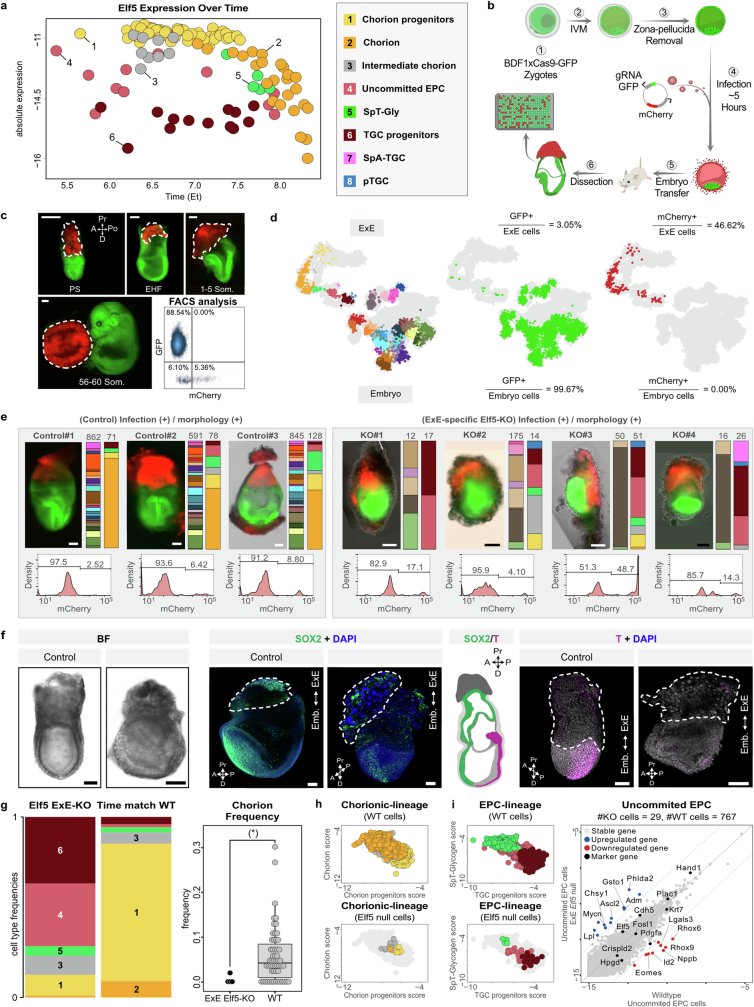
Analysing effects of ExE-specific targeting of Elf5. **a**, WT gene expression of Elf5 among ExE metacells over transcriptional time (E_t_). **b**, Scheme describing the experimental design for exclusive ExE targeting. **c**, Dissected Cas9-GFP post-implantation embryos at four developmental stages, ExE (white dashed line) transduced with a gRNA-GFP with constitutively expressed mCherry. A representative FACS scatter plot obtained from an E7.5 dissected embryo subjected to lentiviral infection prior to implantation. Embryo axes; Anterior (A), Proximal (Pr), Posterior (Po) and Distal (D). **d**, UMAP projection of an E8.0 embryo (single cells coloured by cell state) subjected to lentiviral infection over the WT ExE and embryonic manifolds (grey points). Shown are projections of single cells expressing GFP (green points, middle) and single cells expressing mCherry (red points, right) from the same embryo (left). **e**, Images of analysed control embryos (left panel, Methods, N = 3) and ExE-specific Elf5-KO mutants (right panel, N = 4, infected with gRNAs targeting Elf5). Embryonic (left) and ExE (right) cell-state composition are shown per embryo, together with mCherry distribution as measured by FACS (bottom). **f**, Representative images of control embryos (left, N = 2) and ExE-specific Elf5-KO mutants (right, N = 2) dissected at E7.5. Bright-field (BF, left panel). Representative maximum intensity projection immunofluorescence images, labelling either SOX2 (green, centre) or Brachyury (T, magenta, right) together with DAPI nuclear staining (blue/white in centre and right, accordingly). Scale bar 100 µm; Illustration depicts typical spatial distribution of SOX2 and T in an E7.5 embryo (middle). ExE compartment is marked with a white, dashed line. **g**, Pooled ExE cell-state composition (left panel) for ExE Elf5-KO (left) and time matched WT embryos (right). Boxplot shows the pooled frequency of the chorion among ExE cells in ExE-specific Elf5-KO and time match WT embryos. Centre represents mean. Box bounds indicate the IQR (25th to 75th percentile). Whiskers extend to the minimum and maximum values within 1.5 times the IQR. All the data represents biologically independent samples examined over 5 independent experiments. Each dot represents an individual embryo coloured based on its genotype (as indicated). Wilcoxon rank sum test. (*) - P(t-test) <5%; ns – non significant. **h-i**, Single-cell lineage scores shown for WT (top) and Elf5-KO cells (bottom) from the chorionic lineage (**h**) or EPC lineage (**i**, left panel). Cells are coloured according to their cell state, with background WT cells coloured in light grey (bottom). Bulk differential gene expression between uncommitted EPC cells from Elf5-KO and WT embryos (right panel). Dashed line represent a two-fold difference in expression; solid line represents the x = y diagonal; blue dots – genes showing at least two-fold upregulation in KO; red dots – genes displaying at least two-fold downregulation in Elf5-KO. Black dots – marker genes of EPC lineage. Grey dots – all other genes. Source Data

We next sought to begin delineating the internal signalling driving ExE differentiation on the basis of the new temporal reference model and genetic perturbation. *Elf5* was previously defined as a regulator of TSCs. It was shown through germline knockout (KO) analysis to affect embryonic patterning and preclude derivation of TSCs, with no major effects on early EPC structure^25,26^. Consistently, our temporal model shows high *Elf5* expression distinguishing chorion progenitors from EPC cells as early as E6.0 (Extended Data Fig. 7a). We adjusted a previously developed protocol for ExE-specific genetic perturbations^27^ (Methods and Extended Data Fig. 7b–d), and used gRNAs targeting *Elf5* (coexpressed with mCherry) combined with index sorting and scRNA-seq. We analysed embryos that displayed morphological alterations and robust *Elf5* KO in the ExE (Extended Data Fig. 7e). Immunostaining of ExE *Elf5*-KO mutants coincided with previous observations in germline *Elf5*-KO mutants^25,26^, including defects in the formation of the primitive streak and posterior patterning in the embryo (Extended Data Fig. 7f). Mapping single-cell states in ExE *Elf5*-KO tissues showed that, in comparison to matched WT ExE, mutants lacked differentiated chorion cells (Extended Data Fig. 7g,h). To test the potential effects on other ExE cell states in the absence of *Elf5*, we performed differential expression analysis of EPC mutant and WT cells. This confirmed a generally conserved EPC state but also identified the downregulation of *Rhox6* and *Rhox9* and upregulation of additional genes (Extended Data Fig. 7i). The case of *Elf5* highlights the chorion as a potential regulator of both proximal EPC cells and proximal embryonic lineages^28^, prompting further delineation of its role as a signalling centre. Methodologically, these results showed that we can now examine ExE and embryonic regulation over a standardized time axis and, in concert, use single-cell analysis after ExE-specific perturbation, as well as the chimera assays that we described previously^29^.

## ExE BMP4 over time and lineages

**Fig. 4:**
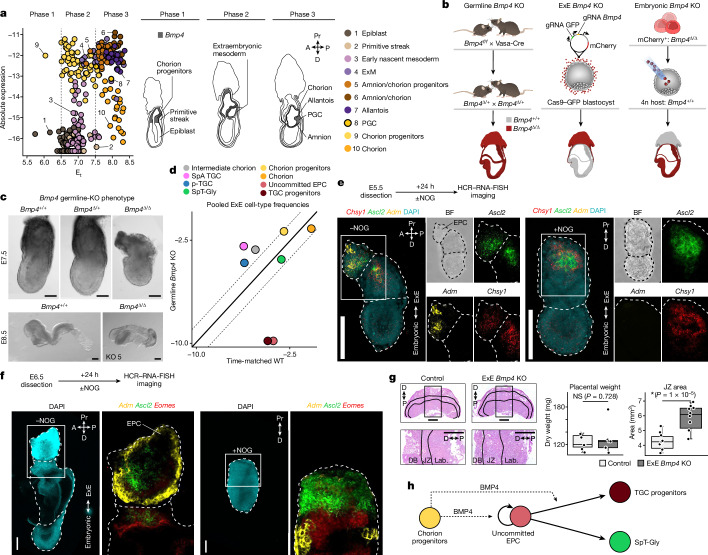
ExE BMP4 is required for proper EPC differentiation. **a**, Metacell expression of *Bmp4* across cell states over time (E_t_), divided into three phases (dashed lines) with schematics showing the potential spatial distribution. **b**, The experimental strategy to generate *Bmp4* KOs in the germline (left), ExE (middle) and embryonic compartment (right). **c**, Bright-field images of representative WT (+/+; *n* = 11 (E7.5), *n* = 3 (E8.5)), heterozygote (Δ/+; *n* = 15 (E7.5), *n* = 12 (E8.5)) and homozygote (Δ/Δ; *n* = 3 (E7.5), *n* = 4 (E8.5)) *Bmp4*-KO genotypes at E7.5 (top) and E8.5 (bottom). All biologically independent samples were examined over six experiments. **d**, The pooled frequency (log_2_ scale) of ExE cell types in germline *Bmp4*-KO mutants over time-matched WT embryos. The solid and dashed lines represent the *x* = *y* diagonal and the twofold difference threshold, respectively. **e**,**f**, Representative images of multiplexed HCR–RNA-FISH analysis of E5.5 (**e**) or E6.5 (**f**) embryos cultured with (*n* = 4 (E5.5), *n* = 8 (E6.5)) or without NOG (*n* = 3 (E5.5), *n* = 9 (E6.5)) for 24 h. The areas indicated by the white boxes are magnified on the right. The white dashed lines mark embryo; the white double-headed arrow shows the embryonic–ExE borders. **g**, Histological sections of E12.5 control (left, *n* = 5) and ExE *Bmp4*-KO (right, *n* = 5) placentas stained with haematoxylin and eosin; all from a single litter. The magnification highlights the expansion of the JZ in the ExE *Bmp4* KO. DB, decidua basalis; lab., labyrinth. The box plots show the placental dry weight (left) and JZ area size (right); the centre line represents the mean, the box limits indicate the interquartile range (25th to 75th percentile) and the whiskers extend to the minimum and maximum values within 1.5× the interquartile range. Statistical analysis was performed using two-tailed *t*-tests; *P < 5%; NS, not significant. **h**, Schematic of the chorion-derived BMP4 effects on EPC differentiation. Scale bars, 1 mm (**g**) and 100 μm (**c**, **e** and **f**). Source Data

**Extended Data Fig. 8:**
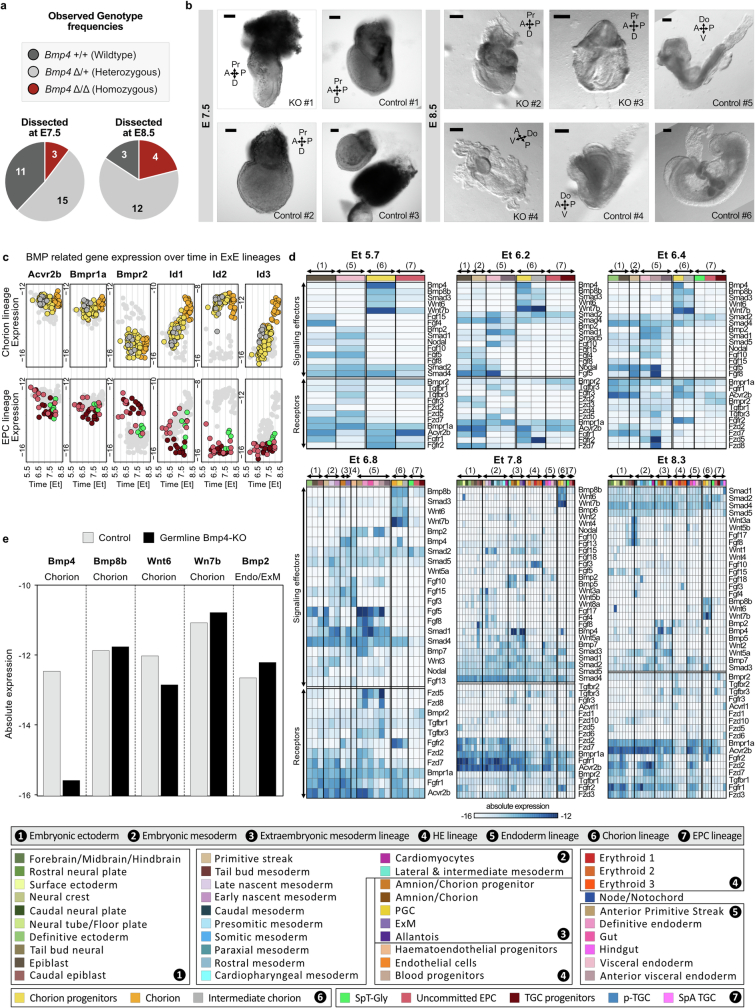
Germline Bmp4-KO statistics and signalling environment over time. **a**, Pie charts of PCR-validated genotypes obtained from crosses of Bmp4(Δ/+) heterozygotes (see Fig. 4b), demonstrating expected Mendelian ratios in E7.5 (top) and E8.5 (bottom). **b**, Bright-field images of analysed control (WT or heterozygous, see Methods) and germline Bmp4-KO mutants from E7.5 (top) and E8.5 (bottom). Embryo axes; Anterior (A), Posterior (P), Proximal (Pr), Distal (D), Dorsal (Do), and Ventral (V). Scale bar 100 µm. **c**, Metacell expression (log2) over E_t_ of selected BMP-related signalling genes in chorion-lineage (top) and EPC-lineage (bottom). Grey points represent all ExE metacells. **d**, Heatmap showing pooled cell type absolute expression (log2) of signalling effectors (top section, associated with TGF-beta, Fibroblast/Epithelial growth factors, WNT, and BMP principal signal transducers) and receptors (bottom section). Each heatmap represents one of the six distinct age-groups, with the average E_t_ for each bin specified in the title. Cell states coloured by their cell state. Lineage relationships of cell states annotated at top as one of seven lineages (see legend below)**. e**, Bulk cell type expression (log2) of BMP/WNT signalling effectors in germline Bmp4-KO and time-matched WT embryos. Source Data

BMP4 is an extensively studied signalling protein driving gastrulation, with previously shown effects on posterior mesoderm, ExM and PGC specification in the mouse^7^. Our model refines quantitatively previous reports, showing that early on *Bmp4* is exclusively expressed from chorion progenitors up to E_t_7.0 (Fig. 4a (phase I)). At this time, BMP4 is induced in early nascent mesoderm, and peaks in expression in the ExM and the amnion and allantois, its differentiated derivatives (Fig. 4a (phase II)). Notably, *Bmp4* expression gradually decreases during chorion and PGC differentiation (Fig. 4a (phase III)). To characterize the impact of the source and time of BMP4 signalling on embryonic and ExE development, we devised a three-way perturbation scheme targeting the gene in the entire embryo (germline *Bmp4*-KO), in the ExE alone (ExE *Bmp4*-KO), or exclusively in the embryo proper (embryonic *Bmp4*-KO) (Fig. 4b). Consistent with previous reports^7^, homozygote germline *Bmp4*-KO embryos (*Bmp4*^*Δ/Δ*^) displayed developmental retardations, including reduced size at E7.5 and no visible allantois at E8.5, compared with heterozygotes (*Bmp4*^*Δ/+*^) and WT littermate controls (Fig. 4c and Extended Data Fig. 8a,b). We performed single-cell transcriptional analysis of five germline *Bmp4*-KO embryos and six control littermates, and compared these with time-matched WT embryos (Methods and Extended Data Fig. 8b). Notably, in the ExE, this analysis identified depletion of uncommitted EPC cells and TGC progenitors together with normal proportions of SpT-Gly and differentiated TGCs (spiral artery and parietal TGCs). By contrast, chorion differentiation seemed not to be affected by the lack of BMP4 (Fig. 4d). Analysis of BMP perception capacity, and no clear evidence for compensation by other ligands (Extended Data Fig. 8c–e), further support a specific role of BMP4 in EPC, but not chorion, differentiation.

**Extended Data Fig. 9:**
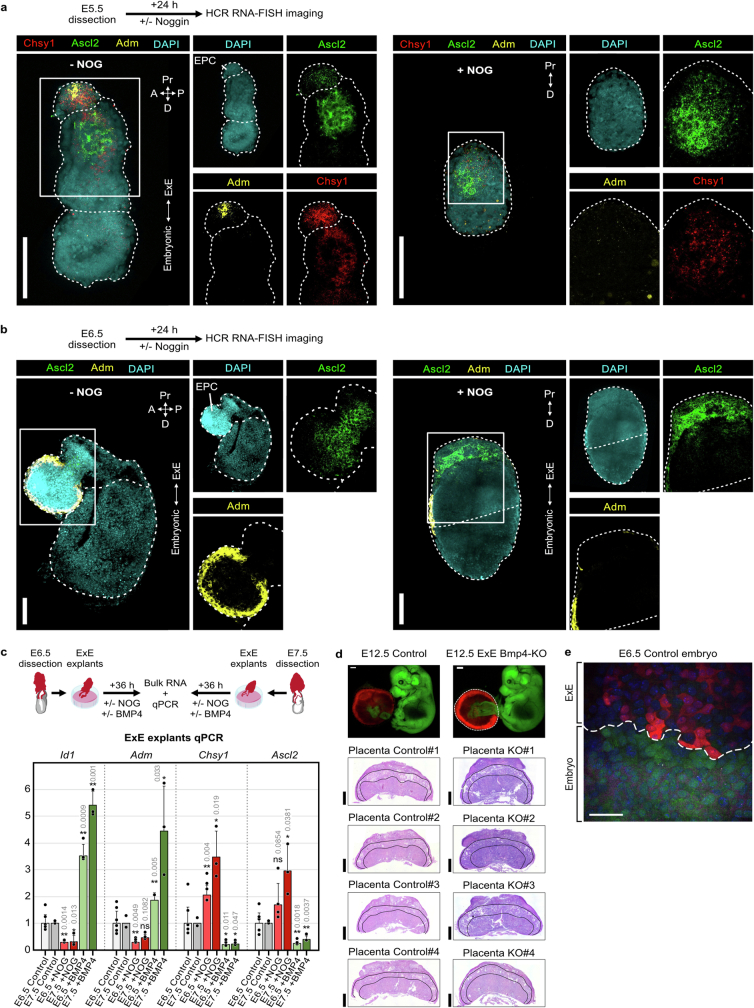
Effects of chorion-derived Bmp4 on ExE differentiation. **a-b**, Representative images of multiplexed HCR-RNA-FISH analysed control embryo (left panel, -NOG) and Noggin treated embryo (right panel, +NOG) after 24 h cultured ex-utero of embryos dissected at E5.5 (**a**) and E6.5 (**b**). Labelling either Chsy1 (red), Ascl2 (green) or Adm (yellow) together with DAPI nuclear staining (blue). Magnified regions (right part in each panel) are highlighted with a white solid line rectangle in left part. Scale bar 100 µm. White, dashed lines mark the embryo borders and Embryonic-ExE border (labelled and oriented with a white double head arrow legend to the right). Embryo axes; Anterior (A), Proximal (Pr), Posterior (P), and Distal (D). **c**, Schematic representation of ExE explant experiment (Methods, top). Relative expression of bulk RNA from E6.5 and E7.5 ExE explants for selected EPC lineage transcription factors in control (E6.5, N = 6: light-grey, E7.5, N = 2: dark-grey) and treated with Noggin (E6.5, N = 4: light-red and E7.5, N = 3: dark-red) or BMP4 (E6.5, N = 3: light-green and E7.5, N = 3: dark-green) explants. All biologically independent samples examined over 2 independent experiments. (*) – q(t-test) <5%; (**) – q(t-test) <1%; ns – non significant, two-tailed. **d**, E12.5 dissected Cas9-GFP embryos (top) infected with mCherry-expressing control vector (Methods, left) or with gRNA against Bmp4 (Methods, right). Representative histological sections of E12.5 control (left panel) and ExE Bmp4-KO (right panel) placentas, stained with Hematoxylin and Eosin (bottom). Black solid line highlights the placental junctional zone region. Scale bar 1 mm. **e**, Embryo-ExE junction magnification of E6.5 dissected Cas9-GFP (green) embryo infected with mCherry-expressing control vector (red) and DAPI nuclear staining (blue), highlighting a mosaic infection. White, dashed line marks the Embryo-ExE border (labelled and oriented to the left). Scale bar 100 µm. Source Data

To evaluate the spatiotemporal effects of BMP4 during EPC differentiation, we cultured E5.5 and E6.5 dissected embryos for 24 h with or without the presence of the BMP antagonist Noggin (NOG). While we detected *Chsy1*- and *Ascl2-*positive cells in NOG-treated embryos, they did not present typical (culture associated) EPC structure and did not induce *Adm* expression, compared with the control embryos (Fig. 4e,f and Extended Data Fig. 9a,b). Treatment of ExE explants with either BMP4 or NOG confirmed reciprocal effects on gene expression (Extended Data Fig. 9c and Supplementary Table 8). To separate the potential effects of embryonic BMP4 from the chorion-derived source, we next targeted *Bmp4* specifically in the ExE lineage (as described above for Elf5; Extended Data Fig. 7b). Histological analysis of highly infected E12.5 placentas identified significant enlargement of the JZ in mutants in comparison to in the controls, without a noticeable effect on the overall placental weight (Fig. 4g, Extended Data Fig. 9d and Supplementary Table 9). Together, our data support the proposal that early BMP4 signalling from chorion progenitors is needed to maintain the potential of uncommitted EPCs, while a later source is important for their specification towards TGCs (Fig. 4h). Note that ExE *Bmp4*-KO embryos developed properly even when dissected at E12.5 (*n* = 54 (control), *n* = 51 (KO); Extended Data Fig. 9d), potentially due to incomplete *Bmp4* ablation (Extended Data Fig. 9e).

## ExE BMP4 affects the mesoendoderm junction

**Fig. 5:**
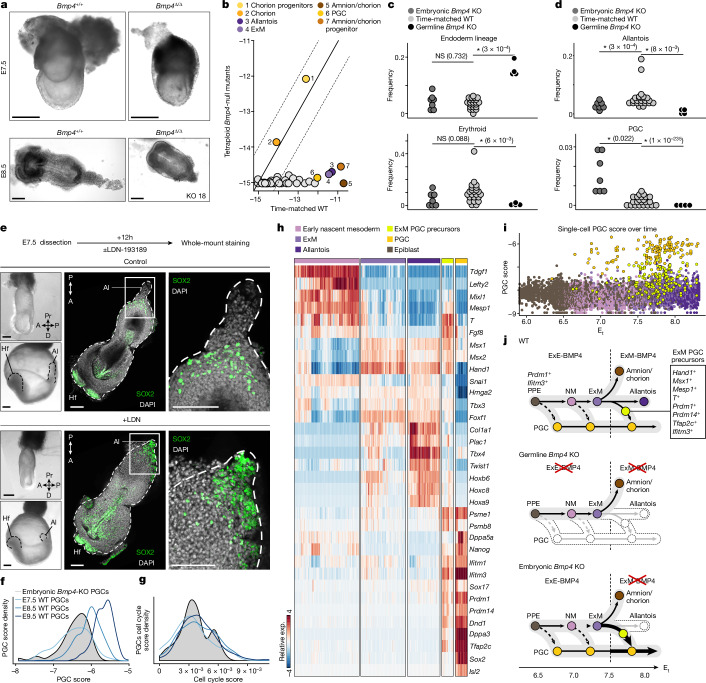
Temporal effects of chorionic and ExM-derived BMP4 on embryonic development. **a**, Representative images of embryonic *Bmp4*-KO mutants (*n* = 18) and controls (*n* = 6). All biologically independent samples were examined over ten experiments. KO 18 (bottom right) indicates knockout mutant number 18. Scale bars, 100 µm. **b**, *Bmp4* expression (log_2_) per cell state between mutant and time-matched WT embryos. The solid and dotted lines represent the *x* = *y* diagonal and the twofold difference threshold, respectively. **c**,**d**, Frequency comparison of endoderm lineage and erythroid (**c**) and allantois and PGC (**d**) cell types per embryo in the *Bmp4*-KO models. Each dot represents an embryo, coloured by genotype. Median frequencies were compared using Wilcoxon rank-sum tests. Low-frequency PGCs *P* values were calculated using two-tailed *χ*^2^ tests (Methods), and were adjusted using the Benjamini–Hochberg procedure. *q < 5%. **e**, 3D images of immunostained control (top, *n* = 3) and LDN-treated (bottom, *n* = 3) embryos dissected at E7.5 and cultured for 12 h. All biologically independent samples were examined over two independent experiments. SOX2 (green) and DAPI (white) are shown. The area indicated by a white box is magnified on the right. Embryo borders are marked by white dashed lines. Images at dissection (top left) and after culture (bottom left) are shown. The dashed lines indicate borders of allantois (Al), head fold (Hf) and embryo. Scale bars, 100 µm. **f**,**g**, The single-cell distribution of different PGC groups over PGC score (**f**; Methods) and cell cycle score (**g**; Methods). **h**, The relative metacell expression of selected cell types, highlighting mutual expression with ExM PGC precursors. **i**, WT single-cell PGC score over time (E_t_). **j**, Simplified schematics of the WT PGC differentiation model depicting changes in cell type frequency observed for WT (top), germline *Bmp4* KO (middle) and embryonic *Bmp4* KO (bottom). ExE-BMP4 (top left) and ExM-BMP4 (top right) indicate the source of BMP4 (separated by dashed vertical lines). The diagram is coloured and annotated on the basis of cell state. Normal differentiation is indicated by the narrow black arrows. The light grey arrows indicate a failure to differentiate. Increase in cell type proportion is shown by bold arrows. The dashed arrows show previously established PGC differentiation dynamics. Source Data

**Extended Data Fig. 10:**
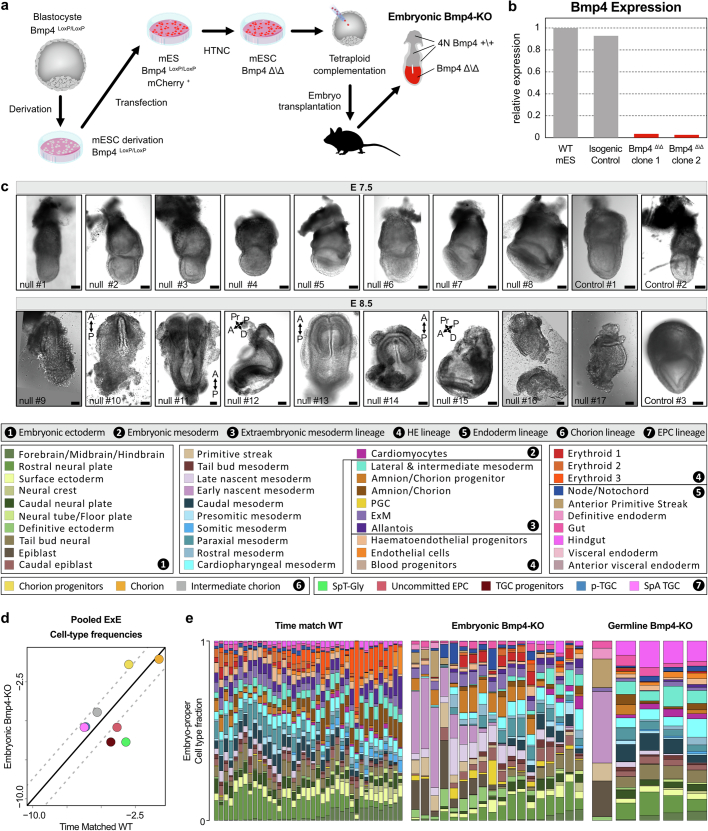
Effects of ExE or embryonic-derived Bmp4 ablation on ExE and embryonic cell specification. **a**, An extended schematic representation of the experimental design used to generate embryonic Bmp4-KO mutants. Derived mES cells used in this experiment were obtained by crossing homozygous Bmp4 LoxP/LoxP embryos, which were also utilized for the germline Bmp4-KO. Followed by HTNC treatment (see Methods). **b**, Real-time PCR analysis showing fold change (ΔΔCt) in Bmp4 expression between the two Bmp4 Δ/Δ clones (red) and one Bmp4 Δ/+ clone (grey, Isogenic control) used in this study, compared to WT (grey). **c**, Bright-field images of analysed control (injected with Isogenic control mES cells, see Methods) and embryonic Bmp4-KO mutants from E7.5 (top) and E8.5 (bottom). Embryo axes; Anterior (A), Proximal (Pr), Posterior (P), and Distal (D). Scale bar 100 µm. **d**, Pooled frequency (in log2 scale) of ExE cell types in embryonic Bmp-KO mutants over time-matched WT embryos. Solid and dashed lines represent x = y diagonal and two-fold difference threshold, accordingly. **e**, Embryo-proper cell type composition (see colour annotation in legend, top) of individual germline Bmp4-KO mutants (N = 5, right), embryonic Bmp4-KO mutants (N = 18, middle) and time-matched WT embryos (N = 39, left). Source Data

We used a tetraploid complementation assay combined with scRNA-seq^29^ (Methods and Extended Data Fig. 10a,b) to compare cell type compositions and gene expression between individual embryos in which *Bmp4* was either depleted exclusively in the embryo proper or in both the embryonic and ExE compartments (Fig. 4b). Consistent with previous reports^8^ embryonic *Bmp4*-KO mutants showed growth retardation and underdeveloped posterior tissues at E7.5 and E8.5 (*n* = 18 (KO); Fig. 5a and Extended Data Fig. 10c). Single-cell sequencing confirmed depletion of *Bmp4* from embryonic derivatives, and WT levels in the ExE (Fig. 5b). Analysis of ExE cell-state compositions in embryonic *Bmp4*-KO mutants showed overall similar frequencies compared with the time-matched WT controls (Extended Data Fig. 10d). This is in contrast to the stronger effects observed in the germline *Bmp4*-KO mutants, narrowing down the impact of BMP4 on EPC development to the ExE chorion lineage.

**Extended Data Fig. 11:**
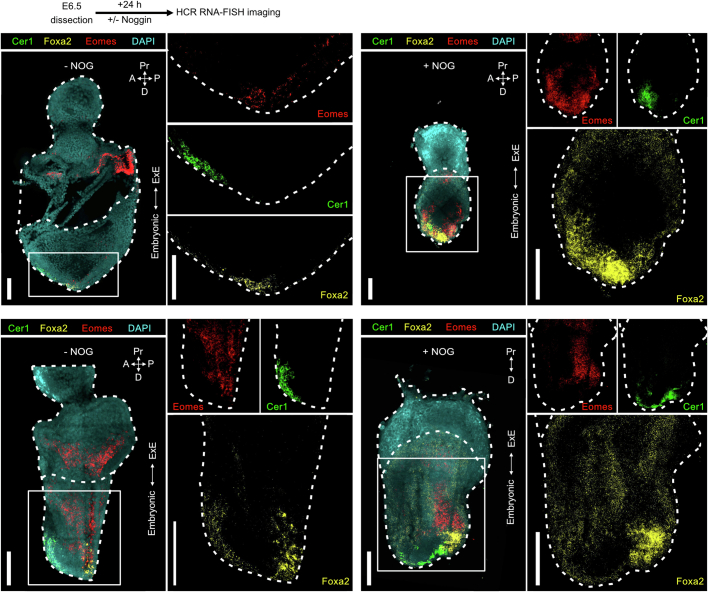
Effects of ExE or embryonic-derived Bmp4 ablation on endoderm specification. Representative images of multiplexed HCR-RNA-FISH analysed control embryo (left side, -NOG, N = 4) and Noggin treated embryo (right side, +NOG, N = 4) after 24 h cultured ex-utero of embryos dissected at E6.5 (schematic representation of experiment in the top). Labelling either Eomes (red), Cer1 (green) or Foxa2 (yellow) together with DAPI nuclear staining (blue). Magnified regions (right part in each panel) are highlighted with a white solid line rectangle in left part. Scale bar 100 µm. White, dashed lines mark the embryo borders and Embryonic-ExE border (labelled and oriented with a white double head arrow legend to the right). Embryo axes; Anterior (A), Proximal (Pr), Posterior (P), and Distal (D).

Next, we systematically compared embryonic cell type compositions after *Bmp4* KO, focusing first on early developmental times in which *Bmp4* is expressed exclusively from the chorion progenitors (Fig. 4a (phase I)). Notably, we identified significant enrichment of endoderm cells in the germline *Bmp4*-KO mutants (but not the embryonic *Bmp4*-KO mutants), alongside marked depletion of all erythroid cells (Fig. 5c and Extended Data Fig. 10e). Consistent with the common notion that NODAL–BMP4 cross-talk is required for proper formation of the primitive streak, our data highlight additional effects on initial mesoendoderm bifurcation. To further test this observation, we stained embryos dissected at E6.5 and cultured ex utero for 24 h in the presence of NOG for early endoderm and primitive-streak markers. Our analysis revealed that all embryos treated with NOG displayed misallocated expression of primitive-streak marker *Eomes*, together with spurious and increased proportions of the anterior primitive-streak marker *Foxa2* (Extended Data Fig. 11), which marks a progenitor cell population of the definitive endoderm^30,31^. This suggests a regulatory role of early chorion-derived BMP4 in balancing early mesoendoderm bifurcation.

## Embryonic BMP4 limits PGC specification

**Extended Data Fig. 12:**
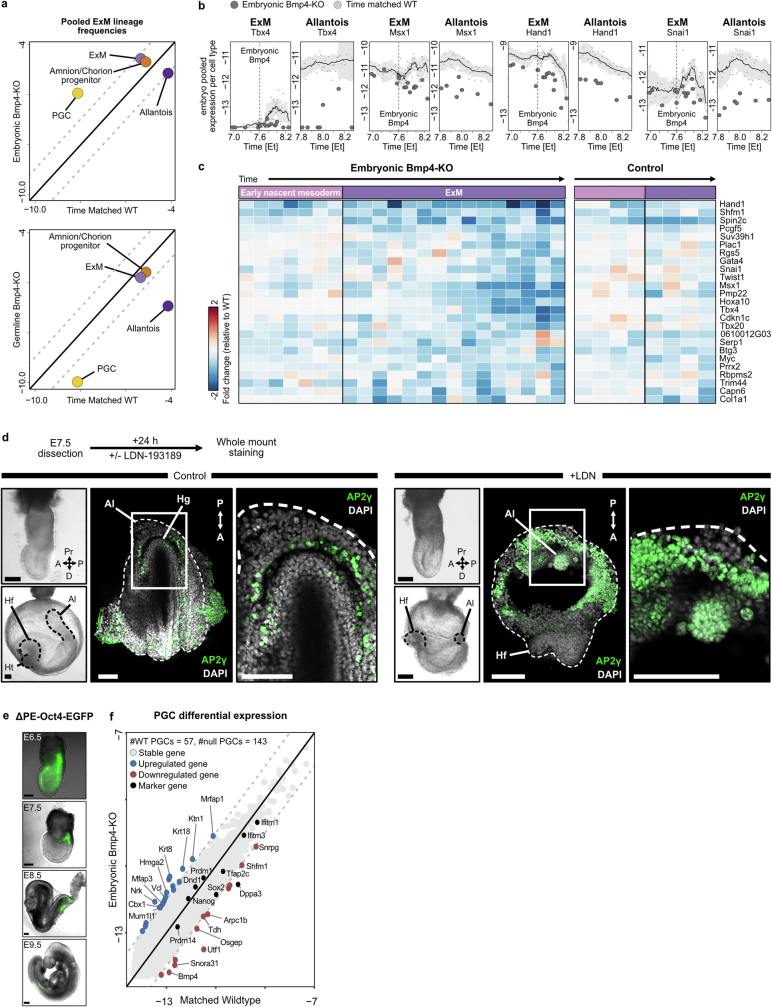
Embryo-derived BMP4 regulates PGC and allantois competing lineage choices. **a**, Pooled frequency (in log2 scale) of ExM cell type derivatives (annotated) in embryonic Bmp-KO mutants (top) or germline Bmp4-KO (bottom) over time-matched WT embryos. Solid and dashed lines represent x = y diagonal and two-fold difference threshold, accordingly. **b**, Gene expression per embryo and cell type for selected genes in ExM (left part) and Allantois (right part). Each dot represents an individual embryo, with WT embryos in small light grey and embryonic Bmp4-KO mutants in big dark grey. Solid black line – WT average expression; grey area – WT average ± moving standard deviation (window size = 13). Initiation of mesoderm *Bmp4* expression marked by dashed line in each ExM expression panel. **c**, Heatmap showing fold change (log2 scale) between pooled cell type expression of embryonic Bmp4-KO mutants (left) and isogenic control (right), relative to time matched WT (see Methods). Within each cell type individual embryos are ordered by E_t_ (early-to-late; left-to-right). **d**, 3D images of immunostained control (top, N = 3) and LDN treated (bottom, N = 3) dissected at E7.5 and cultured for 12 h. AP2C (green) labelling shown with DAPI (white). Magnified region outlined in left panel. Embryo borders marked by white dashed lines. Images at dissection (top left) and after culture (bottom left). Dashed lines indicate the borders of Allantois (Al); head fold (Hf) and embryo. Axes: Anterior (A), Proximal (Pr), Posterior (P), Distal (D). Scale = 100 µm. Embryo positioned from distal-to-proximal view (left) and proximal-to-distal view (right). **e**, Representative images of ∆PE Oct4-GFP reporter embryos at four different developmental stages (dissection day annotated). scale bar 100 µm. Data represents 58 biologically independent samples over seven experiments. **f**, Differential expression between embryonic Bmp4-KO and WT PGCs, stratified by their overall PGC marker expression (see Methods). Solid and dashed lines represent x = y diagonal and two-fold difference threshold, accordingly. Gene trends annotated in legend. Source Data

BMP4 signalling from the ExM was previously implicated in PGC survival and allantois development^8^. Mouse PGCs were speculated to share an ancestral origin with ExM derivatives, originating from a cell population located at the proximal-posterior epiblast^7,32,33^ that induces mesodermal genes such as *T* and *Fgf8*^34–37^. In our data, both germline *Bmp4*-KO and embryonic *Bmp4*-KO mutants had a WT-comparable frequency of ExM and amnion/chorion progenitors (Extended Data Fig. 12a), suggesting that BMP4 is dispensable for these fates. However, while germline *Bmp4*-KO embryos exhibited the expected reduction in both allantois cells and PGCs, ablating *Bmp4* exclusively from the embryo resulted in a marked increase in PGC frequency along with a moderate yet significant reduction in allantois cells (Fig. 5d). Temporal gene expression analysis in the WT model showed that the allantois program initiates in the ExM, after the induction of *Bmp4* in these cells (Extended Data Fig. 12b). Importantly, ablation of the embryonic source of BMP4 resulted in marked repression of *Tbx4*, together with genes encoding additional key ExM TFs, such as *Msx1*, *Hand1* and *Snai1* (Extended Data Fig. 12b,c). Our data are consistent with ExM-derived BMP4 being necessary for promoting ExM differentiation towards allantois programs, but not the amnion fate.

To further examine the function of embryo-derived BMP4 on PGCs and allantois as potentially competing lineage choices, we cultured late-streak-stage embryos in the presence or absence of LDN-193189 (LDN), a small-molecule intracellular inhibitor of BMP signalling^38^ (*n* = 8 (control), *n* = 9 (LDN)). Treated embryos exhibited reduced allantois and developmental delay, overall resembling embryonic *Bmp4*-KO mutants (Fig. 5e and Extended Data Fig. 12d). Immunostaining for SOX2 and AP2γ identified an increase in PGC numbers in the treated embryos compared with the controls. Consistent with a previous observation^8^, this cell population was found at the base of the diminished allantois but also ectopically distributed within this structure. At the migratory stage (24 h after LDN), PGCs were ectopically distributed around most of the posterior axis of the embryo, in stark contrast to the control-treated embryos in which these cells were confined to the posterior hindgut region (Extended Data Fig. 12d).

**Extended Data Fig. 13:**
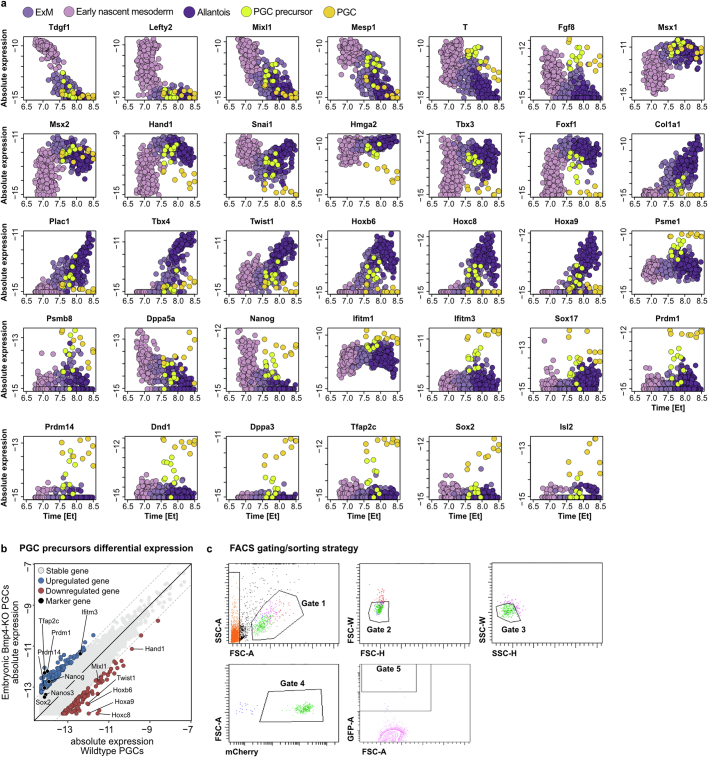
Expression dynamics along the PGC trajectory. **a**, Metacell expression (log2 absolute expression) over transcriptional time (E_t_) for all genes in Fig. 5h. **b**, Pooled differential gene expression between PGC precursor cells from WT and embryonic Bmp4-KO mutants. Dashed line represents a two-fold difference in expression; solid line represents the x = y diagonal; blue/red dots – genes showing at least two-fold upregulation/downregulation in KO (respectively). Black dots – PGC marker genes. Light grey dots – all other genes. **c**, FACS gating and sorting strategy for all single-cell data obtained in this study. ‘Gate 3’ highlights the sorted population for atlas construction, ‘Gate 4’ marks the mCherry-positive population for Embryonic Bmp4-KO data, and ‘Gate 5’ identifies the ΔPE-Oct4-GFP cell population. Source Data

PGCs have been demonstrated to differentiate before gastrulation, within the posterior proximal epiblast region (E5.5–6.0)^39–42^. However, their specification continues up to the early-bud stage (E7.5–7.75)^32,41,43^. To gain further insights into this later PGC specification, we analysed PGCs enriched from embryos of a transgenic mouse line carrying the pluripotency and germ-cell-specific ΔPE-Oct4-eGFP reporter^44^ between E6.5 and E9.5 (Extended Data Fig. 12e). We then compared embryonic *Bmp4*-KO PGCs (dissected at E7.5–8.5) to the resulting enriched PGC trajectory model (Methods). Embryonic *Bmp4*-KO PGCs showed cell-cycle and PGC score distributions and specific gene expression signatures comparable to WT E7.5–8.0 PGCs (Fig. 5f,g and Extended Data Fig. 12f). We next wanted to trace the transcriptional effects in embryonic *Bmp4*-KO mutants leading to overgrowth of the PGC pool. We identified an intermediate transcriptional state within the enriched dataset that concurrently expresses mesoderm and ExM markers (such as *Mesp1, T, Msx1/2* and *Hand1*), together with robust induction of the PGC program (Fig. 5h and Extended Data Fig. 13a). We termed these cells ExM PGC precursors. Notably, in WT embryos, these precursors were prominently observed during E7.5–8.0, but are largely absent after E8.0, simultaneous with the establishment of the allantois program (Fig. 5i). Differential expression of ExM PGC precursors in WT and embryonic *Bmp4*-KO mutants showed downregulation of genes associated with the transition from ExM to allantois (for example, *Hand1*, *Twist1*, *Hoxb6*, *Mixl1* and *Snai1*)^45–48^ and upregulation of PGC-associated markers (such as *Prdm1*, *Prdm14*, *Tfap2c* and *Ifitm3*)^40,49–51^ (Extended Data Fig. 13b). Our data capture early emerging PGCs (E6.5–7.0) but, due to the sparsity of the data, we did not further characterize their molecular makeup (Fig. 5i). In summary, although ExE-derived BMP4 is essential for PGC specification, these results suggest that BMP4 signalling from the ExM at later stages effectively restricts the PGC window of specification by promoting differentiation towards the allantois (Fig. 5j).

## Discussion

We profiled single cells in single embryos to model the parallel differentiation of embryonic and extraembryonic cell ensembles during mouse gastrulation^52,53^. The functional coupling between the embryo and ExE is evident, and its detailed understanding has recently gained a new perspective in stem-cell-derived embryonic models^54–58^ and comparative studies in mammals^59^. ExE differentiation is initiated by early (E5.25) branching into EPC and chorionic lineages during egg cylinder formation. We find a pool of continuously differentiating uncommitted EPC cells up until somitogenesis. The mechanisms regulating the maintenance and differentiation of uncommitted EPC cells are currently unclear. However, the observed transcriptional changes during EPC differentiation, alongside differential response to cues from surrounding neighbouring cells, could be instrumental for future attempts to isolate and culture these multipotent cells in vitro. For example, our data support the notion that maintaining uncommitted EPC cells in culture would require inhibiting the BMP pathway in these cells. In contrast to the EPC niche, the chorion program is initiated through progenitor cells that fully converge into differentiated chorion cells by E_t_8.0, eventually harbouring the labyrinth compartment in the mature placenta^5,20^. Positioned at the ExE–embryo junction, the chorion produces essential developmental signalling and may thereby be tightly linked and contribute not only to embryonic lineages as previously postulated^5^ but also to the neighbouring EPC lineage.

To functionally examine the temporal and multilineage coordination between the embryo and ExE, we combined the time-resolved model with lineage-specific gene perturbations. We adapted assays to separately target genes in embryonic and ExE tissues (together or individually), focusing on BMP4—one of the best-studied signalling factors driving gastrulation at the ExE–embryo interface. Our model sheds light on its crucial time- and context-specific function. We identify that, in the ExE, BMP4 signalling is essential for the differentiation of uncommitted EPC cells and the buildup of the EPC niche. In the embryo, ExE-derived BMP4 promotes balanced mesoendoderm bifurcation, as its loss leads to excess endoderm differentiation. The latter is consistent with the established role of NODAL–BMP4 cross-talk for proper formation of the primitive streak^6^. Early ExE-derived BMP4 signal is also essential for PGC and allantois differentiation^6–8^, although we find that it is dispensable for differentiation of the ExM towards amnion. Ablating or disrupting the later embryonic source of BMP4 perturbs the differentiation of ExM towards allantois and coincides with a marked increase in PGC numbers. In contrast to previous claims^8^, we find that the ExM-derived BMP4 signal is dispensable for PGC transcriptional and cell cycle integrity. This is consistent with a recent report showing that BMP4 is dispensable cell-autonomously for PGCs specification^60^. Instead, the data suggest that ExM BMP4 beyond the late streak stage promotes increased activation of the mesodermal program leading to the differentiation of the ExM towards an allantois fate, disfavouring PGCs. Notably, while initial expression of mesoderm genes is essential for PGC formation, continued expression of these same genes is detrimental, as in *Blimp1*-null embryos^34,42^. These findings suggest a temporally defined control for BMP4 over PGC fate acquisition during gastrulation. First, by inducing cells within the proximal posterior epiblast to adopt a germline fate near the early-streak stage^34,39,41,42^; and, second, in restricting the PGC pool during the final recruitment of ExM PGC precursors. Mechanisms restricting PGC pool size have long been debated^7^. Here we propose that a time-dependent BMP4 response may, at least in part, underlie this phenomenon. Notably, we cannot rule out the possibility that later PGC precursors correspond to a population of ExM cells that fail to establish a PGC program and consequently adopt an ExM fate. Current progress in lineage-tracing technologies holds promise to further shed light on the differentiation dynamics of this intriguing lineage.

Together, the data highlight the importance of timing and tissue synchronization during gastrulation. Even though the identity of many (perhaps most) of the major factors driving this process are known, our analysis will prompt further work towards careful and precise localization of gastrulating cells and signals in space and time. This will be essential for elucidating cell-fate decisions and cell-extrinsic mechanisms regulating the self-organization of embryonic and extraembryonic ensembles. More generally, it will be a crucial step towards transforming cell atlases into dynamic and mechanistic models for development.

## Methods

### Embryo recovery and documentation

All of the animal procedures were approved by the Institutional Animal Care and Use Committee and were performed in strict adherence to Weizmann Institute guidelines. Mice were monitored for health and activity and were given ad libitum access to water and standard mouse chow with 12 h–12 h light–dark cycles. Embryos were collected from timed pregnant immune-competent C57BL/6JRccHsd or Hsd:ICR(CD-1) female mice (obtained from Envigo and mated in house with males of the same strain) between E5.5 and E9.5. Embryos were recovered from their implantation sites using fine forceps, in PBS, while carefully preserving all extraembryonic tissues. The embryos were then washed in PBS and transferred to chilled DMEM (Phenol-red free, GIBCO) supplemented with 10% FBS (Biological Industries) for imaging before dissociation. Phase-contrast images were taken using the Eclipse Ti2 inverted microscope (Nikon) and Zyla sCMOS camera (Andor). Morphological staging and analysis of embryos was conducted as previously described^61^.

### Cell line information

Source of cell lines: all of the cell lines used in this study were generated in-house from a stock of validated V6.5 (C57BL/6×129) background. Authentication of cell lines: the cell lines were authenticated using genotyping PCR to ensure their identity and purity. The genotyping results confirmed the expected genetic background for each cell line. Mycoplasma contamination testing: all of the cell lines were routinely tested for mycoplasma contamination using the PCR-based Mycoplasma Detection Kit (Hylab) before use in the experiments. No mycoplasma contamination was detected in any of the cell lines.

### Trophectoderm-specific genetic manipulation

#### Production of lentiviral vectors

Lentiviral vectors were constructed to produce lentiviruses expressing gRNAs designed to selectively target either *GFP* expression and the third exon of *Bmp4* or the third and fourth exons of the *Elf5* gene locus in the trophectoderm of Cas9–GFP^62^ mouse blastocysts. All gRNAs were selected for minimal off-target effects using the CCTop CRISPR/Cas9 target online predictor (https://cctop.cos.uni-heidelberg.de:8043/)^63^. Introduction of a mega-primer (Supplementary Table 4) that includes gRNAs into a lentivector, constitutively expressing gRNAs scaffolds and a mCherry fluorescent reporter, was carried out by restriction-free cloning as previously described^64^. Recombinant lentiviruses were produced by transient transfection into HEK293T cells, using polyethylene imine (PEI) (PEI linear, Mr 25,000, Polyscience) as previously described^65^, using three envelope and packaging plasmids and one of three viral constructs: (1) pDecko-GFP/mCherry (that is, the control vector), (2) pDecko-Elf5/mCherry or (3) pDecko-Bmp4/mCherry. In brief, infectious lentiviruses were collected at 48 and 72 h after transfection, filtered through 0.45-mm-pore cellulose acetate filters and concentrated by ultracentrifugation at 20,000 rpm for 2 h. Lentiviral supernatant effective titres were determined by infection of HEK293T cells followed by fluorescence-activated cell sorting (FACS) analysis. To validate of *Elf5* and *Bmp4* KO, HEK293T cells were infected with the appropriate lentiviral vector expressing gRNAs targeting *Elf5*/*Bmp4* and mCherry. Infected cells were picked by sterile sorting, subsequently transfected with px330 Cas9 targeting plasmid expressing GFP^66^ and sorted again prior to DNA extraction. Genomic DNA was extracted by PCR-compatible lysis buffer (10 mM Tris, pH 8, 0.45% Triton X-100, 0.45% Tween-20, 0.2 mg ml^−1^ proteinase K). Primers flanking the PAM sequence of each target (Supplementary Table 4) were used for amplifying the genomic segments that included the expected Cas9-mediated DNA editing, and immediately followed by Sanger sequencing (not shown).

#### Mice and lentiviral transduction

Infection of nascent blastocysts was performed using B6D2F1 (C57BL/6xDBA) (Envigo)/Cas9-GFP embryos. In brief, 3–4-week old B6D2F1 female mice were hormone primed by an intraperitoneal injection of pregnant mare serum gonadotropin (PMSG, Vetmarket) followed 46 h later by an injection of human chorionic gonadotropin (hCG, Sigma-Aldrich) and mating with homozygote Cas9-GFP males^62^. Embryos were collected at the zygote stage, and cultured in KSOM medium until the blastocyst stage. For efficient infection of the trophectoderm, zona pellucida was removed in acidic Tyrode’s solution (Sigma-Aldrich)^27^. Next, 15–20 embryos were incubated with lentiviruses, described above, in KSOM for 4–5 h. The transduced blastocysts were washed, and then were transferred into each recipient female generated after mating with vasectomized CD1 males (Envigo); the day of injection was considered to be 2.5 days post coitum. Mice were handled in accordance with institutional guidelines and approved by the Institutional Animal Care and Use Committee (IACUC, The Weizmann Institute of Science).

#### Analysis of genetically manipulated embryos

*Elf5*-KO embryos were collected at E7.5, and *Bmp4*-KO embryos were collected in a time series from E7.5 to 8.5, and all were dissected in ice-cold 1× PBS. Individual mutants were imaged using the Eclipse Ti2 inverted microscope (Nikon) and the Zyla sCMOS camera (Andor) while being maintained in DMEM supplemented with 10% FBS. Embryos positive for mCherry were selected for further morphological and transcriptome analysis (only in *Elf5*-KO embryos).

### Tetraploid complementation assay for generating embryonic *Bmp4* KO

*Bmp4*^*LoxP/LoxP*^ embryonic stem cells were derived from *Bmp4*^*LoxP/LoxP*^ mice^67^ (C57BL/6×129; Extended Data Fig. 11a) using standard embryonic stem cell derivation method. The cells were then treated with recombinant His-TAT-NLS-Cre (HTNC) protein (Addgene plasmid, 13763). For genotyping, individual clones were grown for two passages on gelatin-coated plates to eliminate residual MEF, and RNA was extracted using Direct-zol (Zymo RNA miniPrep, R2052) followed by cDNA production and quantitative PCR (qPCR; Supplementary Table 4). For this study, we used two validated *Bmp4*^*Δ/Δ*^ clones and one isogenic control clone (a *Bmp4*^*+/+*^ HTNC-treated clone). Blastocyst injections were performed using (C57BL/6xDBA) B6D2F1 (Envigo) host embryos. In brief, 3–4-week old B6D2F1 females were hormone primed by an intraperitoneal injection of pregnant mare serum gonadotropin (PMSG, Vetmarket) followed 46 h later by an injection of human chorionic gonadotropin (hCG, Sigma-Aldrich). Embryos were collected at the zygote stage, and cultured in a CO_2_ incubator until the blastocyst stage. For tetraploid complementation, two-cell embryos were fused to one cell using a CF150/F instrument (BLS), by 2 DC square pulses of 30 V 40 ms and 1–2 V AC, in 0.3 M mannitol solution with BSA. On the day of the injection, embryos were placed in M2 medium using a 16-µm-diameter injection pipet (Biomedical Instruments) and a Piezo micromanipulator (Prime Tech); approximately 15 cells were injected into the blastocoel of each embryo. Approximately 20 blastocysts were transferred to each recipient female (CD1 female mice, Envigo); the day of injection was considered to be E2.5. Mice were handled in accordance with institutional guidelines and approved by the Institutional Animal Care and Use Committee (IACUC, The Weizmann Institute of Science).

### Ex utero culture of post-implantation embryos

Pregastrulating embryos were dissected at E5.5, their Reichert’s membrane removed and individually placed into separate wells of 8-well glass-bottom ibiTreat μ-plates (iBidi; 80827/80826) filled with 250 μl of EUCM (consisting of 25% DMEM (GIBCO 11880; includes 1 mg ml^−1^ d-glucose and pyruvate, without phenol red and without l-glutamine) supplemented with 1× GlutaMax (GIBCO, 35050061), 100 U ml^−1^ penicillin–100 μg ml^−1^ streptomycin (Biological industries; 030311B) and 11 mM HEPES (GIBCO, 15630056), plus 50% rat serum (rat whole-embryo culture serum, ENVIGO Bioproducts B-4520) and 25% human umbilical cord blood serum (prepared in-house)). The medium was preheated for an hour in an incubator under 5% CO_2_ at 37 °C. Embryos were cultured statically under 5% CO_2_ at 37 °C. The total volume of the medium was replaced every 24 h, and the embryos were monitored by morphological assessment daily.

### BMP inhibition in ex utero and explant cultures

For ex utero experiments, embryos were meticulously dissected at either E5.5 or E6.5 and processed for ex utero culture as described above. During the 24 h culture period, the embryos were co-cultured with 400 ng ml^−1^ of mouse recombinant Noggin (R&D systems, 1967-NG). Continuous morphological monitoring was conducted, and comprehensive analysis was performed using multiplexed RNA in situ HCR, as described below. In ExE explant cultures, embryos at E6.5 and 7.5 were dissected, and the ExE and EPC were precisely isolated from the embryonic compartment. Subsequent culture procedures followed an established protocol^41^, with the addition of 800 ng ml^−1^ of mouse recombinant Noggin or 1000 ng ml^−1^ mouse recombinant BMP4. After incubation for 24 h, bulk RNA was extracted and purified using the Micro RNA kit (Qiagen, 74004). qPCR was then used to quantify selected markers (primer details are provided in Supplementary Table 4). For temporal depletion of BMP signalling, embryos were dissected at E7.5 and were subjected to ex utero culture as described above. During the 12/24 h culture period, the embryos were co-cultured with 5 µM LDN-193189 (Sigma-Aldrich, SML0559), followed by whole-mount immunostaining.

### Immunostaining

In this study, whole-mount immunostaining was performed as previously described^24^, using the following antibodies: rabbit anti-EOMES (1:100, ab23345, Abcam); rabbit monoclonal anti-Brachyury (D2Z3J) (1:100, Cell Signaling, 81694); mouse anti-KRT7 (1:100, Abcam; ab9021); goat anti-SOX2 (1:100, R&D, AF2018); rabbit anti-TFAP2C (1:100, CST, 2320); donkey anti-rabbit IgG (IgG) (H+L), Alexa 647 (1:250, Jackson ImmunoResearch, 711-605-152); donkey anti-goat IgG (H+L) Alexa 488 (1:250, Jackson ImmunoResearch, 705-545-003); goat anti-mouse IgG1 Alexa Fluor 594 (1:250, Jackson ImmunoResearch, 115-585-205).

### Spatial analysis

Multiplex RNA in situ HCR^22^ was performed according to the manufacturer’s instructions (Molecular Technologies). For sample preparation, embryos were dissected and their Reichart’s membrane removed in M2 medium at consecutive timepoints after implantation until E7.5. Embryos were then washed in cold PBS and fixed in 4% PFA 4 °C overnight. After fixation, the samples were dehydrated on ice in increasing ratios of methanol (Sigma-Aldrich) and PBST (0.1% Tween-20) (Sigma-Aldrich) until freezing overnight in absolute methanol −20 °C. The embryos were then rehydrated in increasing ratios of PBST and methanol on ice, washed in PBST, treated with 10 μg ml^−1^ proteinase K (Thermo Fisher Scientific) and subjected to post-fixation in PFA 4% all at room temperature. For HCR staining, the samples were repeatedly washed with PBST, prehybridized with probe hybridization buffer (Molecular Technologies) for 30 min at 37 °C and then hybridized with probe sets (Molecular Technologies) for different combinations of *Adm*, *Sox2*, *Eomes*, *Fgfr2*, *Ascl2*, *Bmp4*, *Lefty1*, *Fosl1*, *Chsy1*, *Hand1* and *Pcdh12* (16 nM) at 37 °C overnight. Tissues were washed with HCR probe wash buffer (Molecular Technologies), followed by repeated washes in 5× SSCT (5× SSC with a final concentration of 0.1% Tween-20) and incubated with HCR amplifiers (Molecular Technologies) (30 pmol) in amplification buffer (Molecular Technologies) at room temperature overnight. The samples were then washed with 5× SSCT, labelled, mounted and imaged in an eight-well glass bottom/ibiTreat μ-plates (iBidi; 80827/80826). Spatial analysis was conducted using the Leica STELLARIS 8 Spectral confocal microscope and acquired using LAS-X (Leica). Fluorophores were excited by a white light laser and acousto-optical beam splitter. All embryonic specimens were visualized by maximum-intensity projection of their fluorescence signals across focal planes, and their 3D structure was assessed. The presented images were finalized using ImageJ software.

### Flow cytometry

For isolation of single cells for scRNA-seq analysis, embryos were dissociated with 0.25% trypsin-A, 0.02% EDTA (Biological Industries) solution for 5 min at 37 °C and resuspended in DMEM without phenol red (GIBCO) supplemented with 10% FBS (Biological Industries). The samples were run on the FACS Aria-III flow cytometer (BD Biosciences, using BD FACSDiva v.9.0) using the ‘index sort’ option to retain the spectral properties of each individual sorted cell. For samples of ΔPE-Oct4-GFP^44^ obtained after E7.5, we further dissected areas on the basis of the localization of GFP expression. Subsequently, we used index-sorting and enriched for GFP-positive cells. The gating and sorting strategy is shown in Extended Data Fig. 13c. FlowJo v.10.7 was used to generate Extended Data Fig. 7c.

### scRNA-seq analysis

#### 10x Genomics

Cultured embryos were prepared for sequencing at different developmental stages after 2 days in culture: late streak and early head fold, assessed by light microscopy. Six similar embryos were selected for each developmental stage, pooled and dissociated using trypsin-EDTA solution A 0.25% (Biological Industries, 030501B) for 5 min at 37 °C. Trypsin was neutralized with medium that included 10% FBS and cells were washed and resuspended in 1× PBS (calcium and magnesium free) with 400 μg ml^−1^ BSA. Cell suspension was then filtered with a 70 μm cell strainer to avoid cell clumps. Single-cell viability was determined by trypan blue staining, before being diluted to a final concentration of 1,000 cells per μl. scRNA-seq libraries were generated for each pool of embryos separately using the 10x Genomics Chromium v3 system (5,000 cell target cell recovery) and sequenced on the Illumina NovaSeq 6000 platform according to the manufacturer’s instructions.

#### MARS-seq

Single-cell cDNA plate based libraries were prepared as previously reported^68,69^ according to the MARS-seq protocol^70^, following index FACS as described above.

### scRNA-seq data processing

#### 10x Genomics data from ex utero cultured embryos

Raw files were transformed into count matrices using Cell Ranger v.6.1.2 with the default parameters and with the prebuilt Cell Ranger reference package refdata-gex-mm10-2020-A (mm10 genome, GENCODE vM23/Ensemble 98). Cells with less than 2,000 counts, more than 30,000 counts or a high number of counts from mitochondrial genes relative to the number of counts from ribosomal genes were removed, resulting in 9,387 cells from the batch of late streak embryos and 3,916 cells from the batch of head fold stage embryos. For doublet removal, we ran DoubletFinder separately on the two batches, following the best practice workflow of the package. In brief, after creating a Seurat object for each batch^69^, Seurat principal component analysis was performed on the basis of the 2,000 most variable feature genes. For doublet detection using DoubletFinder, we calculated the fraction of artificial nearest-neighbour doublet cells (pANN) for each cell using pN = 0.25 (the relative frequency of artificial doublet cells relative to real cells) and pK = 0.02 for the batch of late streak embryos and pK = 0.01 for the batch of head fold embryos (pK is the relative neighbourhood size for the estimation of pANN). In the case of the late streak stage batch, we removed all cells with pANN > 0.2 (*N* = 1,753); for the batch of head fold stage embryos, we removed all cells with pANN > 0.25 (*N* = 291).

#### MARS-seq

MARS-seq libraries were sequenced using the NextSeq 500 or NovaSeq 6000 system. Reads were processed according to the MARS-seq2.0 protocol^70^ with the same specifications as previously reported^12^ using the STAR aligner for read alignment. Overall, we processed 129,024 wells, including the 40,868 wells of the previous version of the gastrulation atlas.

### Metacell analysis and atlas construction

The basic idea of metacells is to partition cells into small groups of homogeneous cells, thereby removing the sparsity of RNA transcript counts associated with scRNA-seq technologies. Throughout the Article, we used metacell sizes of around 20–100 cells. For each metacell *m*, the absolute expression *e*_*g*,*m*_ of a gene *g* is defined as the total number of transcript counts of this gene among all cells belonging to this metacell, normalized to the total number of counts, that is, ${e}_{g,m}={\sum }_{\{c\in m\}}{N}_{g,c}/({\sum }_{g}{\sum }_{\{c\in m\}}{N}_{g,c})$, where *N*_*g*,*c*_ is the number of the number of transcripts of gene *g* in the cell *c*.

#### Embryonic and extraembryonic WT atlas

To identify feature genes for metacell construction^13^, we selected all genes satisfying a minimal variance over mean (T_vm = 0.1) and coverage threshold (T_tot = 50 and T_top3 = 3). These 1,534 filtered genes were clustered into 120 clusters on the basis of their gene–gene correlation across the manifold. We manually selected and removed clusters enriched with cell-cycle- or stress-related genes, leaving 1,386 feature genes. The final metacell object (Knn = 100, minimal metacell size = 20) contained 983 metacells comprising 67,843 cells, including cells from E4.5 blastocysts.

#### Metacell object of ex utero culture embryos

The initial set of 1,017 feature genes was clustered into 60 groups and cell-cycle- and stress-related groups were removed. The metacell cover (148 metacells, 11,684 cells) was constructed on the basis of the remaining 829 feature genes and using the same parameters as above.

#### Metacell2 object of *Bmp4-*KO embryos

Using the framework of Metacell2 (ref. ^14^), all cells from the *Bmp4*-KO experiment were combined into a single-metacell object, including 6,705 cells from germline *Bmp4*-KO embryos, 11,968 cells from embryonic *Bmp4*-KO mutants, as well as 2,380 cells from control embryos. Metacells were constructed using the default parameters and a target size of 150,000 UMIs per metacell.

### Embryo selection and temporal ordering

In total, cells from 287 individual embryos contributed to the embryonic and extraembryonic WT atlas. This includes cells from 153 embryos used in a previous version of the WT atlas^12^. Moreover, we collected 114 cells (after quality control) from two pooled samples of E4.5 blastocysts (*n* = 11 embryos). We selected 251 embryos with a sufficient number of embryonic cells for assigning them a developmental time (shown in Fig. 1c). The frequencies of ExE cell types were estimated on the basis of a cohort of 83 embryos (Extended Data Fig. 2b–d). This cohort included all of the embryos for which we collected the whole ExE tissue during dissection, for which we have a bright-field image to assign them a morphological stage and for which we obtained at least 10 ExE cells after quality control and filtering. Embryos with a sufficient number of embryonic cells (251 embryos) were temporally ordered as previously reported^12^. In brief, using the embryonic cells of an embryo, we calculate an embryo-embryo similarity matrix (Extended Data Fig. 1e) that quantifies the transcriptional similarity between two embryos. Using the similarity matrix, we compute a global goal function for each possible embryo order. Embryos are initially ordered on the basis of their morphology and then are reshuffled; each reshuffling is accepted if it improves the goal function. The final temporal ranks of each embryo are translated into developmental times (denoted as E_t_) by a spline interpolation of the nominal time of collection of each embryo versus its inferred transcriptional rank.

### Network flow model construction

In a previous publication^12^ we introduced a network flow model that enabled us to reconstruct cellular differentiation trajectories during mouse gastrulation along the single-embryo single-cell transcriptome atlas. To infer cellular trajectories on the extended gastrulation atlas, temporally ranked embryos were grouped into 16 time bins (Fig. 1c). The network flow model for all embryonic cells was computed as previously reported. Logistic distances between metacells were calculated using the default parameters. For the estimated proliferation rate of each metacell, we interpolated between the default rate (3.5 divisions per day) and no cell division on the basis of each cell’s expression of cell-cycle-related genes. We used the same values for all additional network flow parameter as in the in the original network flow model of mouse gastrulation.

### Differential expression statistical analysis

The expression of a gene between two groups of cells is compared using a *χ*^2^ test on the number of UMIs from that gene. If ${N}_{g}^{1}$ and ${N}_{g}^{2}$ are the number of UMIs per gene *g* and *N*^1^, *N*^2^ the total number of UMIs per group, we compute for each gene the *χ*^2^ statistic between the two two-dimensional vectors $({N}_{g}^{1},{N}^{1}-{N}_{g}^{1})$ and $\left({N}_{g}^{2},{N}^{2}-{N}_{g}^{2}\right)$. If multiple hypotheses are tested, *P* values are corrected using the Benjamini–Hochberg method.

### Cell cycle scores

For each cell, its synthesis phase (S phase) and mitosis phase (M phase) score is the total number of UMIs from a list of respective marker genes, divided by the total number of UMIs of the cell. The M phase marker genes are as follows: *Mki67*, *Cenpf*, *Top2a*, *Smc4*, *Ube2c*, *Ccnb1*, *Cdk1*, *Arl6ip1*, *Ankrd11*, *Hmmr*, *Cenpa*, *Tpx2*, *Aurka*, AB349069, *Kif4*, *Kif2c*, *Bub1b*, *Ccna2*, *Kif23*, *Kif20a*, *Sgol2a*, *Smc2*, *Kif11*, *Cdca2*, *Incenp* and *Cenpe*. The S phase marker genes are as follows: *Pcna*, *Rrm2*, *Mcm5*, *Mcm6*, *Mcm4*, *Ung*, *Mcm7*, *Mcm2*, *Uhrf1*, *Orc6* and *Tipin*.

### Single-cell scores

Given a list of genes (referred to as cell-state markers) *G*_*m*_, the single-cell score corresponding to a particular cell is calculated as the sum of counts for all genes in a given cell-state markers *G*_*m*_, divided by the total sum of expression counts for all genes in the cell.

### EPC lineage analysis

Let *e*_*g*,*m*_ be the absolute expression for each gene *g* and all metacells *m* from the EPC lineage (uncommitted EPC, TGC progenitor, SpT-Gly), normalized to the total number of counts per metacell, and let *le*_*g*,*m*_ = log_2_(*e*_*g*,*m*_ + 10^−5^). We selected all variable genes that (1) pass a threshold of minimal expression in at least one of the metacells, that is:

$$\mathop{\min }\limits_{m}l{e}_{g,m}\, > \,-13$$

and that (2) pass a threshold on the difference between the highest and smallest expression in a metacell:

$$\mathop{\max }\limits_{m}l{e}_{g,m}-\mathop{\min }\limits_{m}l{e}_{g,m} > 2.$$

A complete list of these genes is provided in Supplementary Table 1. For further clean-up, we filtered only genes that show a minimal difference in their maximal expression among TGC progenitors and SpT-Gly metacells:

$$|\mathop{\max }\limits_{m\in \text{TGC}}l{e}_{{gm}}-\mathop{\max }\limits_{m\in \text{SpT}-\text{Gly}}l{e}_{{gm}}| > 1.5.$$

We observed that the large majority of the filtered genes followed one of the following behaviours: (1) high expression in TGC progenitor metacells compared to SpT-Gly and vice versa; (2) high expression in early metacells and low expression in late; or (3) low expression in early metacells and high expression in late ones. The filtered genes were therefore grouped into five clusters (arguing that this number should be enough to capture the above behaviours; Extended Data Fig. 3a) using *k*-means on the relative expression profiles, $l{f}_{g,m}\,=l{e}_{g,m}-{\text{mean}}_{m}l{e}_{g,m}$. Genes from clusters 1 and 2 were used for the SpT-Gly score and genes from the clusters 4 and 5 were used for the TGC progenitor score. For pseudotime kinetics of gene expression, we fitted a principal curve to the joint distribution of Uncommitted EPC, SpT-Gly and TGC progenitor scores for all cells from the EPC lineage (Extended Data Fig. 3b) and divided the curve into 12 bins (Fig. 2a,b).

### Chorion lineage analysis

Variable genes were filtered for all metacells from the chorion lineage (chorion progenitors, chorion) using the same parameters as for the EPC lineage (Supplementary Table 3). As in the EPC lineage, we also only filtered genes with

$$|\mathop{\max }\limits_{m\in \text{Chorion prog.}}l{e}_{{gm}}-\mathop{\max }\limits_{m\in \text{Chorion}}l{e}_{{gm}}| > 1$$

and clustered their relative expression into 5 clusters (Extended Data Fig. 4a). Genes from clusters 1 and 2 were used for chorion progenitor score and genes from clusters 4 and 5 for the chorion score. Gene expression kinetics (Fig. 2c,d) is shown along the principle curve fitted to the joint distribution of chorion progenitor and chorion scores for each chorion and chorion progenitor cell along 12 bins (Extended Data Fig. 4b).

### Cell type annotation of cells from embryos with genetic manipulations

Cells from EXE *Elf5*-KO, all *Bmp4-*KO models and control embryos were annotated with a cell type from the WT atlas as previously described^14,29^. For each experiment, we constructed a joint metacell *k*-nearest neighbours similarity graph consisting of query cells from the KO embryos and WT atlas cells. For each query and each atlas cell, we sampled the empirical distribution of cell types from atlas cells among its 100 nearest neighbours. Each query cell is matched with an atlas cell (and annotated with its cell type) on the basis of matching the empirical cell type distribution of the query cell with the best-correlated cell type distribution among the atlas cells. Query cells with less than ten atlas cells among their nearest neighbours were assigned with an atlas cell type by computing the cell-metacell correlation cor_*g*_(log_2_(*u*_*g*,*c*_ + 1), log_2_(*e*_*g*,*m*_ + 10^−5^)) for each atlas metacell over the list of feature genes and using the cell type of the best-correlated atlas metacell.

### Developmental timing of *Elf5*-KO and *Bmp4*-KO embryos

Given a temporal order of embryos from the WT atlas, query embryos were assigned a best-matching WT rank as previously reported^14,29^: using the joint metacell *k*-nearest neighbours similarity graph of query cells and atlas cells, each query cell is annotated with the temporal rank of the nearest neighbour atlas cell (that is, the temporal rank of the embryo this cell belongs to). In a similar way, we resample for each atlas cell a temporal rank by assigning to it the rank of the nearest neighbour cell from a different atlas embryo. As a result, we obtain for each embryo a distribution of sampled temporal ranks of the cells belonging to the embryo. Each query embryo is mapped onto a WT rank by computing the correlation between the cumulative distributions over ranks between the query embryo and the WT embryos and using the temporal rank of best-correlated WT embryo.

### Atlas projection and cell type annotation of cells from ex utero cultured embryos

Each cell from ex-utero-cultured embryos was projected onto the WT atlas by matching its expression profile with the best-matching atlas metacell profile: Let *u*_*g*,*c*_ be the single-cell gene expression matrix for all cells *c*> from ex utero cultured embryos and *e*_*g*,*m*_ the WT atlas gene expression per metacell *m* for all genes *g* from a set of 581 feature genes. On the basis of the cell-metacell correlation matrix

$${\text{cor}}_{g}({\log }_{2}{(u}_{g,c}+1),{\log }_{2}({e}_{g,m}+{10}^{-5}))$$

each cell is matched with its best-correlated metacell and annotated with the cell type of the metacell.

Similarly, each metacell from ex utero cultured embryos is matched with its best-correlated WT atlas metacell using the correlation matrix

$$\text{cor}({\log }_{2}{(u}_{g,k}+1),{\log }_{2}({e}_{g,m}+{10}^{-5}))$$

where *u*_*g*,*k*_ is the metacell expression matrix per gene *g* and metacell *k* from the ex utero cultured embryos.

### Cell type frequency comparison between ex utero cultured and WT embryos

To compare the distribution of cell states in ex utero cultured embryos to the WT distribution, we first computed for each batch of ex utero cultured embryos (late streak and late head fold) a best-matching developmental time, thereby correcting for potential differences in the mean developmental time of the two groups. Ex utero cultured embryos were assigned an average developmental time using for each ex utero cell *c* the best-matching atlas metacell *m*(*c*). For a batch *b* of ex utero embryos, let ${p}_{m}^{b}$ be the number of ex utero cells for which *m*(*c*) = *m* normalized to the total number of cells, that is, the number of cells that project to that atlas metacell normalized by the total number of cells from that batch. Vice versa, for each WT embryo *e* (indexed here by their temporal rank 1, 2, ..., 235), let ${p}_{m}^{e}$ be the number of cells from that embryo in the metacell *m* normalized to the total number of cells from that embryo. As the number of cells per metacell is low for single embryos, we use instead of ${p}_{m}^{e}$ the averaged frequency of cells ${\bar{p}}_{m}^{e}={\sum }_{f,|f-e|\le w}{p}_{m}^{f}$ for comparison with ${p}_{m}^{b}$ (over a time window *w*). More precisely, each ex utero batch was matched with that WT embryo *e* for which thedistance between the two distributions ${p}_{m}^{b}$ and ${\bar{p}}_{m}^{e}$ was minimal, that is,

$$\text{matched WT rank}\,=\,{{\rm{argmin}}}_{e}\,|{p}_{m}^{b}-{\bar{p}}_{m}^{e}|.$$

For the batch of late streak embryos we used a window size *w* = 20 and for the batch of late head fold embryos window size *w* = 5.

### Differential expression analysis for *Bmp4*-KO embryos

#### Identification of batch-related genes and metacell–metacell projection

We created a metacell object of all cells from the *Bmp4* experiment. Metacells from the *Bmp4* experiment were projected onto the WT atlas using the framework of MCProj^14^. This approach enables us to match each query metacell *m* and its expression profile ${e}_{g,m}^{{\rm{query}}}\,$ (*g* is a gene) with a corresponding matched WT atlas profile ${e}_{g,m}^{{\rm{proj}}}$. Both profiles represent the relative absolute expression, that is, $1={\sum }_{g}{e}_{g,m}^{{\rm{query}}}={\sum }_{g}{e}_{g,m}^{{\rm{proj}}}$. To screen for batch-related genes, we computed the log fold changes between pseudo-bulk query and atlas expression profiles for all cells *c* from a cell type*t* and experimental condition *b*, that is:

$${lfc}_{g,t}^{b}={\log }_{2}\left(\frac{1}{{N}_{b,t}}\sum _{c\in (b,t)}{e}_{g,m(c)}^{{\rm{query}}}+\varepsilon \right)-{\log }_{2}\left(\frac{1}{{N}_{b,t}}\sum _{c\in (b,t)}{e}_{g,m(c)}^{{\rm{proj}}}+\varepsilon \right)$$

where *m*(*c*) is the metacell *m* of the cell *c*, *N*_*b*,*t*_ is the number of cells from cell type *t* and experimental condition *b* and *ε* = 5 × 10^−5^. There are four different experimental conditions: (1) homozygous *Bmp4*^*Δ/Δ*^ cells from germline *Bmp4*^*Δ/+*^ mating (characterized on the basis of total low levels of BMP4 per embryo), (2) heterozygous *Bmp4*^*Δ/+*^ or *Bmp4*^*+/+*^ cells from germline *Bmp4*^*Δ/+*^ mating (normal levels of BMP4), (3) cells from embryonic *Bmp4*-KO mutants (tetraploid) and (4) isogenic control cells from injected (tetraploid) WT embryos (Extended Data Fig. 11a,b). We subsequently clustered all differentially expressed genes (genes *g* for which $\mathop{\max }\limits_{t,b}|{{lfc}}_{g,t}^{b}| > 0.8$ and that pass a minimal expression threshold) into ten clusters. Genes from clusters displaying differential expression among the control cells were classified as lateral genes.

#### Differential expression per embryo and cell type in the ExM lineage

For each query embryo *e*, we computed the bulk expression per cell type *t* and gene *g*, ${f}_{g,e,t}^{q}$ and the bulk expression per cell type of time-matched WT embryos ${e}_{g,e,t}^{{\rm{WT}}}$. To analyse the effect of embryonic *Bmp4*-KO on the ExM lineage, we selected all of the expression profiles from the cell types early nascent mesoderm, ExM and allantois and from the control and embryonic *Bmp4*-KO mutants that contained at least ten cells per cell type and embryo. We then filtered all of the genes for which (1) $\mathop{\max }\limits_{e,t}| {\log }_{2}(\,{f}_{g,e,t}^{q}+\varepsilon )\,-{\log }_{2}(\,{f}_{g,e,t}^{{\rm{WT}}}+\varepsilon )| \ge {\log }_{2}3$, that is, displaying a threefold change between embryonic *Bmp4*-KO and matched WT cells in at least one of the cell types and one of the embryos; and (2) passing a threshold on minimal expression in one of the conditions, that is, $\mathop{\max }\limits_{e,t}(\,{f}_{g,e,t}^{q},{f}_{g,e,t}^{{\rm{WT}}}\,)\ge 1\times 1{0}^{-4}$. After removing lateral genes (see the previous paragraph), this resulted in 46 genes, which were subsequently clustered into three groups. Cluster 2 is shown in Extended Data Fig. 12c. Genes of clusters 1 and 3 are listed in Supplementary Table 5.

#### Identification and differential gene expression of ExM PGC precursors

Let *e*_*g*,*m*_ be the absolute expression for each gene *g* and all metacells *m* from the PGCs, allantois and ExM, normalized to the total number of counts per metacell, and let *le*_*g*,*m*_ = log_2_(*e*_*g*,*m*_ + 10^−5^). We selected all variable genes that (1) pass a threshold of minimal expression in at least one of the metacells, that is:

$$\mathop{\min }\limits_{m}l{e}_{g,m}\, > \,-13$$

and that (2) pass a threshold on the difference between the highest and smallest expression in a metacell:

$$\mathop{\max }\limits_{m}l{e}_{g,m}-\mathop{\min }\limits_{m}l{e}_{g,m} > 4.$$

A complete list of these genes is provided in Supplementary Tables 6 and 7. For further clean-up, we filtered only genes that show a minimal difference in their maximal expression among PGCs and Allantois or ExM metacells:

$$|\mathop{\max }\limits_{m\in \text{PGC}}l{e}_{{gm}}\,-\,\mathop{\max }\limits_{m\in \text{Allantois},\text{ExM}}l{e}_{{gm}}\,| > 1.5.$$

As in the ectoplacental cone lineage, the filtered genes were therefore grouped into five clusters using *k*-means on the relative expression profiles, *lf*_*g*,*m*_ = *le*_*g*,*m*_ − mean_*m*_*le*_*g*,m_. Genes from clusters 1 were used to calculate the PGC score, and genes from clusters 3, 4 and 5 were used to calculate the allantois–ExM score. On the basis of these scores, we identified a population of 10 metacells consisting of 164 cells that are probably PGC precursors. These metacells were labelled as ExM PGC precursors on the basis of their intermediate-level PGC score and their early developmental time (Fig. 5i–k). Of the 164 cells, 32% originated from embryonic *Bmp4*-KO mutants, 52% from ΔPE-Oct4-GFP embryos (that is, PGC enrichment assay, see above) and the remaining 16% were from the atlas. To investigate differential gene expression, we compared PGC precursor cells from embryonic *Bmp4*-KO mutants with those from WT embryos (Fig. 5k) as described above. We also compared PGC cells from embryonic *Bmp4*-KO mutants to matched WT cells, which were a subset of cells from each group that was stratified by its PGC score (Extended Data Fig. 12d).

### Estimated and sampled number of EXE cells per age group

The representativeness of cells in the ExE was rigorously validated using a dual-pronged approach. Initially, the ExE-to-embryonic cell ratio was computed for each age group, offering a quantitative assessment of the relative abundance of ExE cells throughout the developmental stages. Complementing the ratio calculation, nucleus count data from a previous study^12,14^ were used to estimate ExE cell numbers across diverse developmental stages. The datasets were harmonized by systematically pairing each embryo in the current study with a corresponding embryo from the previous dataset, aligning them on the basis of morphological stages. The integrated datasets enabled a thorough analysis of the congruence between the sampled ExE cells in the current study and the anticipated cell numbers at each timepoint. This comparison, visually depicted in Extended Data Fig. 2d, highlights the close correspondence observed between the sampled and expected ExE cell numbers, affirming the robustness of the used cell sampling methodology.

### Reporting summary

Further information on research design is available in the Nature Portfolio Reporting Summary linked to this article.

## Online content

Any methods, additional references, Nature Portfolio reporting summaries, source data, extended data, supplementary information, acknowledgements, peer review information; details of author contributions and competing interests; and statements of data and code availability are available at 10.1038/s41586-024-07937-5.

## Supplementary information

Reporting Summary

Supplementary Table 1 EPC lineage marker genes. Absolute expression (log_2_) of variable differentially expressed genes within the EPC lineage (see Fig. 2 and Extended Data Fig. 3) annotated with their corresponding cluster.

Supplementary Table 2 Specialized TGCs marker genes. Absolute expression (log_2_) of differentially expressed genes within specialized TGCs annotated with their corresponding cell type mark.

Supplementary Table 3 Chorion lineage marker genes. Absolute expression (log_2_) of variable differentially expressed genes within the Chorion lineage (see Fig. 3 and Extended Data Fig. 4) annotated with their corresponding cluster.

Supplementary Table 4 gRNA and Primers. Primers and guide RNAs used for this study.

Supplementary Table 5 Differential expression in ExM of embryonic *Bmp4*-KO. List of genes that showed significant differential expression between WT ExM and embryonic *Bmp4*-KO ExM (Extended Data Fig. 12b,c).

Supplementary Table 6 ExM/Allantois marker genes. List of genes showing enrichment within the ExM/Allantois (Fig. 5f,h,i and Extended Data Fig. 13a).

Supplementary Table 7 PGC marker genes. List of genes showing enrichment within the PGCs (Fig. 5f,h,i and Extended Data Fig. 13a).

Supplementary Table 8 ExE explants BMP4/NOG RT–qPCR results. RT–qPCR results for explants cultured for 24/36 h in the presence or absence of BMP4/NOG (Extended Data Fig. 9c).

Supplementary Table 9 ExE *Bmp4*-KO E12.5 placental data. Measurements of placentas isolated from E12.5 ExE *Bmp4*-KO embryos (Extended Data Fig. 9d).

## Source data

Source Data Fig. 1

Source Data Fig. 2

Source Data Fig. 4

Source Data Fig. 5

Source Data Extended Data Fig. 1

Source Data Extended Data Fig. 2

Source Data Extended Data Fig. 3

Source Data Extended Data Fig. 4

Source Data Extended Data Fig. 5

Source Data Extended Data Fig. 6

Source Data Extended Data Fig. 7

Source Data Extended Data Fig. 8

Source Data Extended Data Fig. 9

Source Data Extended Data Fig. 10

Source Data Extended Data Fig. 12

Source Data Extended Data Fig. 13

## Data Availability

All sequencing data supporting the conclusions of this study have been meticulously archived and are publicly accessible through the NCBI Gene Expression Omnibus (GEO). These data are catalogued under the GEO Series accession number GSE267870. This ensures comprehensive availability and transparency of the data supporting our research findings. Source data are provided with this paper.

## Code availability

All custom scripts used in this study have been made openly accessible and can be found at GitHub (https://github.com/tanaylab/EmbExe). Moreover, these scripts have been deposited for permanent archiving and citation at Zenodo^71^ (10.5281/zenodo.11240229). This ensures transparency and reproducibility of the computational methods employed in our research.
