# Supplementary material for: Temporal BMP4 effects on mouse embryonic and extraembryonic development
Source: Nature. 2024 Sep 18;634(8034):652–61. doi: 10.1038/s41586-024-07937-5 (PMC11485214; doi:10.1038/s41586-024-07937-5)
Supplement: Supplementary file 1 — Reporting Summary [file 41586_2024_7937_MOESM1_ESM.pdf]

nature portfolio

Corresponding author(s): Yonatan Stelzer, Amos Tanay

Last updated by author(s): Jun 9, 2024

Reporting Summary

Nature Portfolio wishes to improve the reproducibility of the work that we publish. This form provides structure for consistency and transparency in reporting. For further information on Nature Portfolio policies, see our Editorial Policies and the Editorial Policy Checklist.

Statistics

For all statistical analyses, confirm that the following items are present in the figure legend, table legend, main text, or Methods section.

n/a	Confirmed	
☐	☒	The exact sample size (*n*) for each experimental group/condition, given as a discrete number and unit of measurement
☐	☒	A statement on whether measurements were taken from distinct samples or whether the same sample was measured repeatedly
☐	☒	The statistical test(s) used AND whether they are one- or two-sided<br>*Only common tests should be described solely by name; describe more complex techniques in the Methods section.*
☐	☒	A description of all covariates tested
☐	☒	A description of any assumptions or corrections, such as tests of normality and adjustment for multiple comparisons
☐	☒	A full description of the statistical parameters including central tendency (e.g. means) or other basic estimates (e.g. regression coefficient) AND variation (e.g. standard deviation) or associated estimates of uncertainty (e.g. confidence intervals)
☐	☒	For null hypothesis testing, the test statistic (e.g. *F*, *t*, *r*) with confidence intervals, effect sizes, degrees of freedom and *P* value noted<br>*Give P values as exact values whenever suitable.*
☒	☐	For Bayesian analysis, information on the choice of priors and Markov chain Monte Carlo settings
☒	☐	For hierarchical and complex designs, identification of the appropriate level for tests and full reporting of outcomes
☐	☒	Estimates of effect sizes (e.g. Cohen's *d*, Pearson's *r*), indicating how they were calculated

Our web collection on statistics for biologists contains articles on many of the points above.

Software and code

Policy information about availability of computer code

Data collection	Cells were sequenced using Illumina NextSeq 500 and NovaSeq 6000. Flow cytometry data were collected using the BD FACSDiva™ Software v9.0.
Data analysis	All custom scripts have been made available at:<br>https://github.com/tanaylab/EmbExe<br>and deposited at:<br>https://doi.org/10.5281/zenodo.11240229<br><br>Raw files were transformed into count matrices using Cell Ranger 6.1.2 or STAR 2.7.11a<br>using mm10 genome, GENCODE vM23/Ensemble 98<br>Count matrices to metacell objects using Metacells 0.9.5-dev - Single-cell RNA Sequencing Analysis.<br>For FACS plot FlowJo v.10.7 was used.

For manuscripts utilizing custom algorithms or software that are central to the research but not yet described in published literature, software must be made available to editors and reviewers. We strongly encourage code deposition in a community repository (e.g. GitHub). See the Nature Portfolio guidelines for submitting code & software for further information.

Data

Policy information about availability of data

All manuscripts must include a data availability statement. This statement should provide the following information, where applicable:

- Accession codes, unique identifiers, or web links for publicly available datasets
- A description of any restrictions on data availability
- For clinical datasets or third party data, please ensure that the statement adheres to our policy

All sequencing data that support the findings of this study have been deposited in the National Center for Biotechnology Information Gene Expression Omnibus (GEO) and are accessible through the GEO Series accession number GSE267870 at: https://www.ncbi.nlm.nih.gov/geo/query/acc.cgi?acc=GSE267870)

Research involving human participants, their data, or biological material

Policy information about studies with human participants or human data. See also policy information about sex, gender (identity/presentation), and sexual orientation and race, ethnicity and racism.

Reporting on sex and gender	N/A
Reporting on race, ethnicity, or other socially relevant groupings	N/A
Population characteristics	N/A
Recruitment	N/A
Ethics oversight	N/A

Note that full information on the approval of the study protocol must also be provided in the manuscript.

Field-specific reporting

Please select the one below that is the best fit for your research. If you are not sure, read the appropriate sections before making your selection.

☒ Life sciences ☐ Behavioural & social sciences ☐ Ecological, evolutionary & environmental sciences

For a reference copy of the document with all sections, see nature.com/documents/nr-reporting-summary-flat.pdf

Life sciences study design

All studies must disclose on these points even when the disclosure is negative.

Sample size	To capture the full repertoire of cell states, multiple embryos were sampled individually throughout the investigated timeline. Genetically manipulated embryos and embryos from earlier developmental stages, where the total number of cells per embryo is limited, were repeatedly sampled to enrich the number of cells analyzed. Transcriptome analysis of embryos grown in vitro was conducted by pooling embryos from similar morphological stages and comparing them to age-matched WT embryos.<br><br>Sample sizes were chosen to ensure comprehensive analysis and representation of the various cell states. No formal statistical methods were used to predetermine sample size; instead, our approach relied on repeated sampling and pooling to achieve sufficient cell numbers for robust transcriptome analysis. This strategy provides a rationale for the chosen sample sizes, ensuring they are adequate for capturing the diversity of cell states and developmental stages under investigation.
Data exclusions	Lentiviral infected embryos that showed no fluorescent signal, by fluorescent microscopy or by the subsequent FACS analysis, as well as embryos with suffering from poor viability were discarded from the analyzed data. Cells from scRNA-seq with less than 2000 or more than 12000 UMIs were excluded from downstream analysis.
Replication	Cells were sampled from individually sorted embryos, with each embryo representing an independent experiment. The experiments were replicated multiple times, and all attempts at replication were successful. All experimental replication details are provided within the manuscript figure legends to ensure reproducibility.
Randomization	Randomization was not relevant to this study because the focus was on capturing the full repertoire of cell states at various developmental stages. Each embryo was treated as an independent sample, and the primary aim was to analyze the intrinsic variability within and across these stages, rather than comparing treatment groups where randomization would be necessary to reduce bias.<br><br>However, for the ex utero experiments involving embryos treated with LDN BMP4 or Noggin, randomization was applied. Embryos were staged based on their morphology, and experiments were initiated with embryos at the same developmental stage, with random selection used to ensure unbiased sampling.

Blinding	Blinding was applied to experiments involving embryos treated ex utero with LDN BMP or Noggin. In these cases, embryos were staged based on their morphology, and all experiments began with embryos at the same developmental stage. Embryos for these experiments were picked randomly to ensure unbiased selection and to minimize potential bias in the results. For the other experiments, blinding was not necessary as the primary focus was on analyzing developmental stages and cell states within individually sorted embryos, relying on objective measurements that reduce the potential for bias.

Reporting for specific materials, systems and methods

We require information from authors about some types of materials, experimental systems and methods used in many studies. Here, indicate whether each material, system or method listed is relevant to your study. If you are not sure if a list item applies to your research, read the appropriate section before selecting a response.

Materials & experimental systems

n/a	Involved in the study
☐	☒ Antibodies
☐	☒ Eukaryotic cell lines
☒	☐ Palaeontology and archaeology
☐	☒ Animals and other organisms
☒	☐ Clinical data
☒	☐ Dual use research of concern
☒	☐ Plants

Methods

n/a	Involved in the study
☒	☐ ChIP-seq
☐	☒ Flow cytometry
☒	☐ MRI-based neuroimaging

Antibodies

Antibodies used	Rabbit Anti-Eomes (1:100, ab23345,Abcam)<br>Rabbit monoclonal anti-Brachyury (D2Z3J) (1:100, Cell Signaling Cat# 81694)<br>Mouse anti-KRT7 (1:100, Abcam; ab9021)<br>Goat polyclonal anti-Sox2 (1:100, R&D Cat# AF2018)<br>Anti-Rabbit i2, i1,i4 B2,B1, B4 initiator-labeled; Molecular Instruments<br>Donkey Anti-Rabbit IgG (IgG) (H+L), Alex 647 (1:250, Jackson ImmunoResearch, 711-605-152)<br>Donkey Anti-Goat IgG (H+L) Alexa 488 (1:250, Jackson ImmunoResearch, 705-545-003)<br>Goat Anti-Mouse IgG1 Alexa Fluor 594 (1:250, Jackson ImmunoResearch, 115-585-205)<br>Rabbit Anti-TFAP2C (1:100, CST, #2320)
Validation	We adhered to manufacturer guidelines for all antibodies used in this study. Prior to experiments, each antibody underwent testing using wildtype ICR mouse embryos at all required stages, across three different concentrations, to determine the optimal working concentration. Immunofluorescence and HCR experiments were conducted with contemporary technical controls to further validate their efficacy and specificity.

Eukaryotic cell lines

Policy information about cell lines and Sex and Gender in Research

Cell line source(s)	293T HEKs were purchased from ATCC<br>All cell lines used in this study were generated in-house from a stock of validated V6.5 (C57BL/6x129) background or derived directly from natural embryos as explicit in the Methods section.
Authentication	The mES cell lines were authenticated via genotype PCR to confirm their identity and purity, with results confirming their expected genetic background. Additionally, the genome integrity of all mES cells was further validated using common virtual karyotyping, as provided within the code. HEK 293 cells were expanded from a validated stock (Merck, 85120602) to maintain experimental consistency and reliability.
Mycoplasma contamination	All cell lines were routinely tested for mycoplasma contamination using the PCR-based Mycoplasma Detection Kit (Hylabs, KI5034I) prior to use in experiments. No mycoplasma contamination was detected in any of the cell lines.
Commonly misidentified lines<br>(See ICLAC register)	not relevant.

Animals and other research organisms

Policy information about studies involving animals; ARRIVE guidelines recommended for reporting animal research, and Sex and Gender in Research

Laboratory animals	7-8 weeks old B6 abd CD-1 females were crossed with males to isolate post-implantation embryos.<br>3-4 weeks old B6D2F1 (Envigo) females were injected with pregnant mare serum gonadotropin followed by human chorionic

	gonadotropin before being mated with B6-CAS-9-GFP male mice. Blastocysts were transferred into Hsd:ICR(CD-1) female mice (Envigo) which had been mated with vasectomized Hsd:ICR(CD-1) males (Envigo) two days after the vaginal plug was found.
Wild animals	This study did not involve wild animals.
Reporting on sex	All cell lines used were validated males using PCR
Field-collected samples	This study did not involved field-collected samples.
Ethics oversight	All animal procedures were approved by the Institutional Animal Care and Use Committee of the Weizmann Institute of Science and were performed in strict adherence to Institutional guidelines.

Note that full information on the approval of the study protocol must also be provided in the manuscript.

Plants

Seed stocks	N/A
Novel plant genotypes	N/A
Authentication	N/A

Flow Cytometry

Plots

Confirm that:

☒ The axis labels state the marker and fluorochrome used (e.g. CD4-FITC).

☒ The axis scales are clearly visible. Include numbers along axes only for bottom left plot of group (a 'group' is an analysis of identical markers).

☒ All plots are contour plots with outliers or pseudocolor plots.

☒ A numerical value for number of cells or percentage (with statistics) is provided.

Methodology

Sample preparation	Dissociation of isolated post-implantation embryos followed by index-FACS sorting.
Instrument	BD FACSAria III
Software	BD FACSDIVA, FlowJo
Cell population abundance	The purity of post-sort fractions was evaluated by assessing the abundance of relevant cell populations through analysis of specific markers or characteristics using flow cytometry or microscopy. Purity was quantified as the percentage of cells expressing desired markers or exhibiting expected characteristics relative to the total sorted cell population. Samples were randomly selected from dissociated mouse embryos, and statistical analysis was conducted to compare expected versus observed sample sizes (refer to Extended Data Figures 1C and 2D).
Gating strategy	The FACS gating strategy began by excluding debris using the first gate on FSC/SSC. Subsequently, doublets were eliminated using additional gating on FSW/FSH and SSW/SSH parameters. Single cells intended for indexed sorting into scRNA-seq processes were then sorted into barcoded 384-well plates. The complete gating strategy, including 'for atlas construction, mCherry-positive cells in Embryonic Bmp4-KO data, and for ΔPE-Oct4-GFP cells, is detailed in Extended Data Figure 13c.

☒ Tick this box to confirm that a figure exemplifying the gating strategy is provided in the Supplementary Information.
